# Welan Gum, a Bacterial Polysaccharide, Promotes Maize Seedling Growth Through TOR and MAPK Signaling Pathways

**DOI:** 10.3390/gels12080724

**Published:** 2026-08-14

**Authors:** Yulin Fu, Haoran Chen, Shuwen Wang, Wenhui Han, Lin Shen, Wenhui Ma, Xiaoxiao Gao, Wudeng Wang, Fang Yu

**Affiliations:** 1 School of Biological Engineering, Dalian Polytechnic University, Dalian 116034, China; 250240860000229@xy.dlpu.edu.cn (Y.F.); chr15375437721@163.com (H.C.);; 2 Shanghai Key Laboratory of Plant Functional Genomics and Resources, Shanghai Chenshan Botanical Garden, Shanghai 201620, China; 3 Research Institute of Photonics, Dalian Polytechnic University, Dalian 116034, China

**Keywords:** welan gum, maize, plant growth-promoting effects, TOR-MAPK crosstalk

## Abstract

Welan gum, a high-molecular-weight exopolysaccharide from *Sphingomonas* sp., exhibits exceptional rheological stability and robust hydrogel properties, emerging as a highly promising agricultural biomaterial. In this study, we show that the application of welan gum promotes maize seedling growth by enhancing nitrogen use efficiency via the coordinated transcriptional reprogramming of nitrogen regulators and transporters. Mechanistically, welan gum activates the TOR pathway and the MAPK cascade, leading to the phosphorylation of ZmS6K and the terminal MAPK ZmMPK3-1. Notably, TOR inhibition reduces ZmMPK3-1 phosphorylation, demonstrating that TOR activity is indispensable for MAPK activation. Furthermore, the TOR components ZmTOR and ZmLST8 physically interact with ZmMPK3-1, and ZmLST8 independently enhances ZmMPK3-1 kinase activity, revealing a multilayered regulatory mechanism triggered by welan gum. In addition, welan gum upregulates MPK3-targeted defense genes, effectively reconciling the plant growth–defense trade-off. Elucidating this TOR–MAPK crosstalk provides the mechanistic basis for translating welan gum into sustainable gel-based biofertilizers. Collectively, this study uncovers a cooperative signaling network that underpins welan gum-mediated growth promotion, establishing the strong potential of PGPR-derived polysaccharide hydrogels as sustainable, functional biofertilizers for modern agriculture.

## 1. Introduction

Rhizosphere-colonizing soil microbes capable of enhancing plant growth and resilience are collectively termed plant growth-promoting rhizobacteria (PGPR). PGPR exhibit a wide array of beneficial activities for plants, encompassing crucial functions such as regulating hormonal balances to ensure optimal plant development [1], managing nutritional balances by making essential nutrients more available, inducing systemic resistance against a wide spectrum of plant pathogens, and solubilizing minerals and nutrients locked in the soil matrix to enhance plant uptake and overall growth [2,3]. Moreover, PGPR engage in complex ecological interactions within the rhizosphere and the bulk soil, displaying both synergistic and antagonistic relationships with other microorganisms [4,5,6]. These interactions can either amplify the beneficial effects on plant health or mitigate the impact of harmful microorganisms, thereby indirectly contributing to accelerated plant growth and improved resilience [7]. By fostering beneficial microbial communities and suppressing pathogenic populations, PGPR contribute to a more favorable environment for plant growth. In the context of sustainable agriculture, PGPR are fundamental for enhancing crop resistance to diseases, improving tolerance to environmental stresses [8], and optimizing nutrient utilization.

PGPR form biofilms composed of extracellular polymeric substances (EPSs), natural, high-molecular-weight polymers, containing polysaccharides, proteins, and extracellular DNA (eDNA) [9,10]. In our earlier work, we studied *Sphingomonas* sp. strain Y503, which produces exopolysaccharides and enhances both growth and defense mechanisms in *Catharanthus roseus*, a medicinal plant valued for its terpenoid indole alkaloids (TIAs) [11]. As soil and aquatic Gram-negative bacteria, *Sphingomonas* species enhance plant vigor and soil conditions [12,13]. Their characteristic exopolysaccharides, termed sphingans, include variants such as gellan (S-60), welan (S-130), rhamsan (S-194), and diutan (S-657). These compounds feature a tetrasaccharide backbone (glucose-glucuronic acid-glucose-rhamnose/mannose) with diverse side chain modifications including glucosyl, rhamnosyl, mannosyl, and acetyl groups [14].

EPS help plants withstand various environmental stresses, including drought, salinity, temperature extremes, and exposure to heavy metals [15]. These bacterial polysaccharides enhance rhizosphere conditions by promoting soil aggregation, shaping microbial communities, facilitating nutrient uptake, and modulating plant physiological processes [16]. We isolated and characterized the exopolysaccharide from *Sphingomonas* sp. Y503 as welan gum, an anionic polysaccharide with acetyl and L-rhamnosyl or L-mannosyl side groups [17]. Previous research showed that welan gum enhances carbon and nitrogen assimilation in rice seedlings [18], while our pot experiments demonstrated significant growth promotion in maize seedlings. Unlike bacterial strains, whose effectiveness varies with environmental conditions, polysaccharides offer consistent benefits through their biodegradability, non-toxicity, water solubility, and physicochemical stability. Therefore, understanding welan gum’s growth-promoting mechanisms will advance its agricultural applications.

Microbial polysaccharides influence plant signaling networks, including phytohormone transduction, mitogen-activated protein kinase (MAPK), and reactive oxygen species (ROS) pathways, thereby stimulating plant growth [19,20]. Our findings demonstrate that welan gum-induced growth promotion involves both target of rapamycin (TOR) and MAPK pathways, which regulate plant development and environmental responses. TOR, a conserved serine/threonine kinase within the phosphoinositide 3-kinase (PI3K)-related family, acts as a central metabolic sensor that coordinates diverse cellular processes [21]. In parallel, MAPK signaling operates through a three-tiered kinase cascade comprising MAPK Kinase Kinase (MAPKKK, MEKK/MAP3K), MAPK Kinase (MAPKK, MKK/MEK/MAP2K), and MAPK (MPK) [22]. These modules form a phosphorylation relay connecting upstream receptors to downstream targets, including protein kinases, transcription factors, cytoskeletal components, and metabolic proteins [23]. Elucidating how TOR and MAPK pathways mediate signals triggered by welan gum to enhance plant growth will offer valuable insights into the function of microbial polysaccharides in promoting plant development.

The extensive use of synthetic fertilizers in agriculture significantly contributes to environmental chemical pollution [24,25]. Plant growth-promoting rhizobacteria (PGPR)-derived polysaccharides, such as welan gum, present an eco-friendly alternative by enhancing nutrient utilization efficiency and forming hydrogels that improve soil conditions. Although these compounds show considerable promise for sustainable agriculture, their commercial applications remain limited because the molecular mechanisms underlying their growth-promoting effects are largely unknown. Elucidating these mechanisms is essential to move from empirical observations to rational product development, enabling the reliable translation of such polysaccharides into commercial gel-based fertilizers.

## 2. Results and Discussion

### 2.1. Physicochemical and Rheological Characterization of Welan Gum

**Figure 1:**
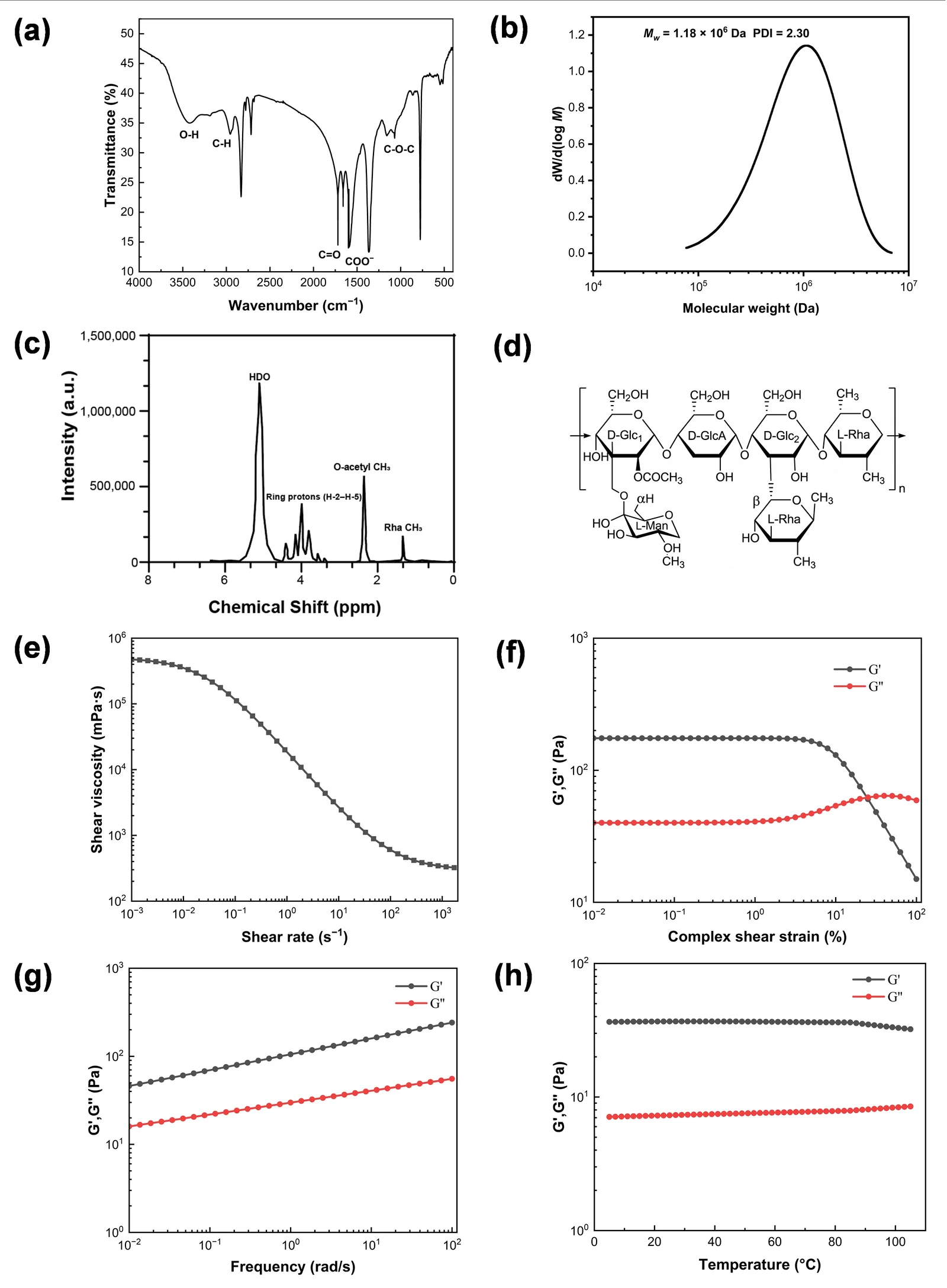
Physicochemical and structural characterization of the extracted welan gum. (**a**) FT-IR spectrum recorded in the range of 4000–400 cm^−1^. (**b**) Molecular weight distribution determined by GPC, showing the weight-average molecular weight (M_w_) and polydispersity index (PDI). (**c**) ^1^H NMR spectrum (600 MHz, D_2_O) of welan gum. (**d**) Chemical structure of the welan gum repeating unit. (**e**) Steady-state shear flow behavior of the welan gum hydrogel (0.5% *w*/*v*) showing shear-thinning behavior. (**f**) Amplitude strain sweep (10 rad/s, 25 °C) identifying the linear viscoelastic region (LVR). (**g**) Dynamic frequency sweep (1.0% strain, 25 °C) showing the frequency-dependent mechanical spectra. (**h**) Oscillatory temperature sweep (10 rad/s, 1.0% strain) demonstrating the thermal stability of the hydrogel network. Symbols: G′ (storage modulus, gray) and G″ (loss modulus, red).

The extracted EPS from *Sphingomonas* sp. Y503, obtained at a yield of 25.42 ± 0.85 g/L, was initially characterized for its physicochemical properties, structural identity, and rheological behavior to assess its potential for agricultural use. The purified EPS exhibited a high carbohydrate content with low residual protein and nucleic acid levels. Its elemental composition was determined to be 39.25 ± 0.52% C, 6.41 ± 0.19% H, and 0.58 ± 0.09% N, with no detectable sulfur (details in Table S1). The FTIR spectrum exhibited typical absorptions of welan gum [26], including the characteristic O-acetyl ester band at 1720 cm^−1^ (Figure 1a). Specifically, key absorption bands are assigned as follows: ~3400 cm^−1^ (O-H stretching, hydroxyl groups), 2920 cm^−1^ (C-H stretching, alkyl groups), 1720 cm^−1^ (C=O stretching, O-acetyl substituents), 1600 cm^−1^ and 1410 cm^−1^ (asymmetric and symmetric stretching, COO^−^), and ~1040 cm^−1^ (C-O-C stretching, glycosidic linkages). Gel permeation chromatography (GPC) revealed a weight-average molecular weight of 1.18 × 10^6^ Da (PDI = 2.30) (Figure 1b). This high molecular weight, together with the O-acetyl groups, constitutes a key structural basis for the polymer’s functional properties. The extended polymer backbone promotes chain entanglement, while the O-acetyl substituents sterically hinder ordered crystallization, which favors the formation of a highly hydrated three-dimensional network [27]. The ^1^H NMR spectrum displayed characteristic chemical shifts for anomeric protons and side-chain methyl groups (δ 1.2–5.5 ppm) (Figure 1c). Key chemical shifts are assigned as follows: δ 5.0–5.5 (anomeric protons of sugar residues); δ 3.0–4.2 (ring protons of sugar moieties, H-2 to H-5); δ 2.0–2.2 (methyl protons of O-acetyl groups); δ 1.2–1.3 (methyl protons of L-rhamnose, Rha CH_3_, typically a doublet). Due to the high viscosity of the welan gum aqueous solution, the spectrum was acquired at an elevated temperature of 333 K (60 °C) to improve spectral resolution. All these spectral features are consistent with the reported structural characteristics of welan gum (Figure 1d) [28,29].

Macroscopic rheological measurements further confirmed its favorable hydrogel properties. The EPS hydrogel exhibited pronounced shear-thinning behavior (Figure 1e) and maintained structural integrity within the linear viscoelastic region (Figure 1f). This shear-thinning pseudoplasticity can be attributed to the physical nature of the crosslinks, allowing easy dispersion under high shear and rapid recovery of the elastic network under static conditions. Frequency sweep tests revealed a solid-like elastic network, in which the storage modulus (G′) consistently exceeded the loss modulus (G″) across the measured frequency range (Figure 1g). Notably, this three-dimensional network showed high thermal stability, with G′ and G″ remaining nearly constant from 5 °C to 105 °C (Figure 1h), indicating that gelation is driven by dense physical entanglements and hydrogen bonding rather than by thermo-reversible associations [30,31]. These rheological characteristics are expected to confer direct functional advantages in the rhizosphere. The shear-thinning property enables efficient delivery via spraying or irrigation, and the rapid recovery of a solid-like elastic network in the soil can help retain moisture and nutrients around the root zone [32]. High thermal stability ensures that the hydrogel maintains its structural integrity under fluctuating field conditions, providing a persistent matrix at the root-soil interface. Beyond serving as a physical barrier against abiotic stress, these material properties could therefore establish a stable root-hydrogel microenvironment that facilitates prolonged biochemical interactions [33,34]. Together, these results indicate that the extracted EPS is welan gum with a highly stable structural framework, providing a material basis that could support its role in agricultural applications.

### 2.2. Welan Gum Exhibits Plant Growth-Promoting Effect on Maize Seedlings

**Figure 2:**
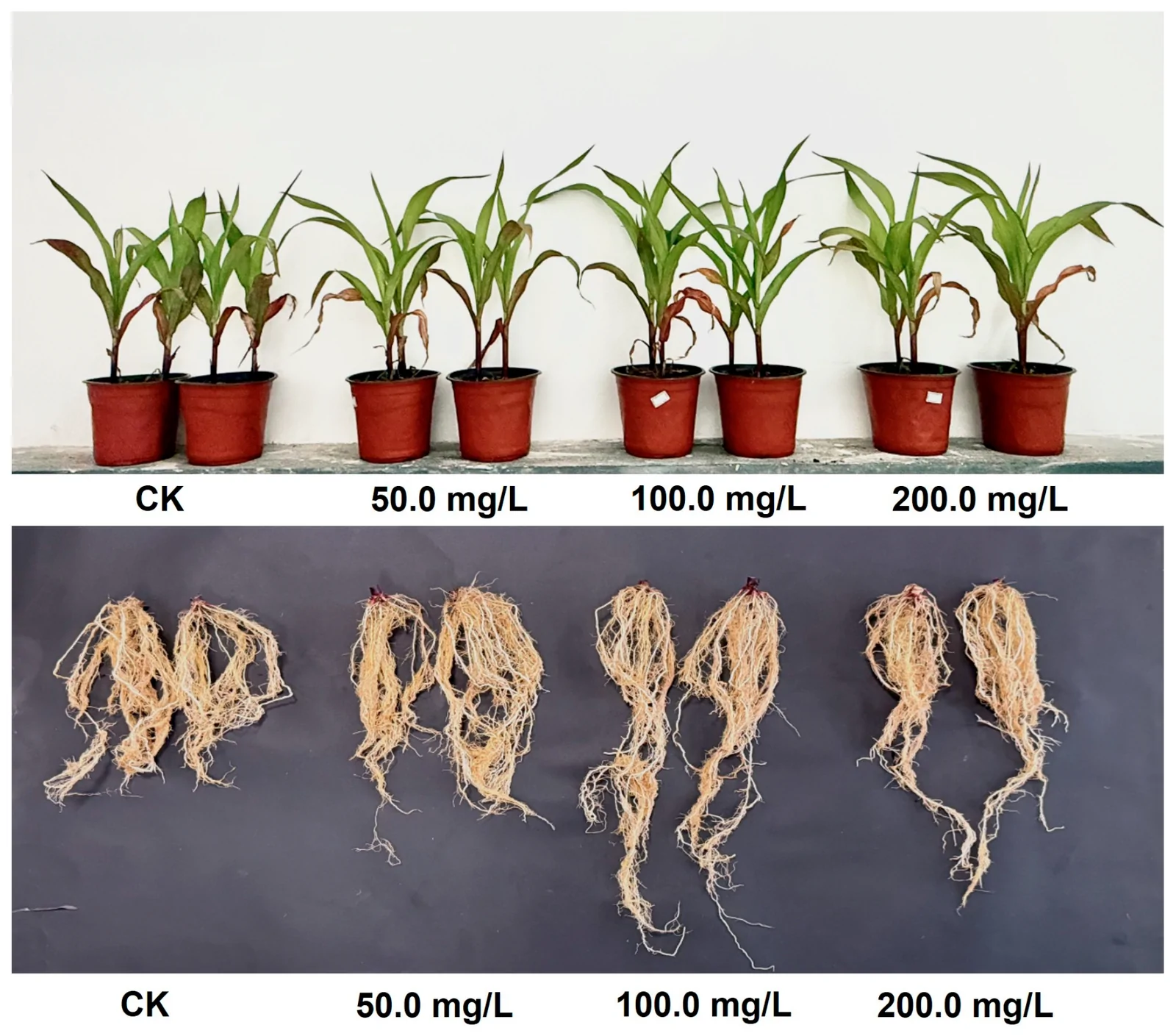
Welan gum promotes maize seedling growth. Maize seedlings were grown in soil-filled pots and treated via root irrigation for 10 days. The treatments consisted of normal irrigation (CK) and welan gum solutions at concentrations of 50.0, 100.0, and 200.0 mg/L for 10 days.

Owing to its distinct rheological properties, welan gum has been widely applied in industrial sectors, yet its effects in plant growth promotion remain poorly characterized. Therefore, a pot experiment was conducted to evaluate welan gum’s effects on maize seedling development. The application of welan gum significantly influenced the vegetative growth of maize seedlings in a concentration-dependent manner (Figure 2, Table 1). The 100.0 mg/L treatment resulted in the most pronounced enhancements across all measured parameters. Specifically, plant height, shoot fresh weight, root fresh weight, and leaf area were highest at this concentration, showing increases of approximately 24.9%, 52.02%, 51.8%, and 23.2%, respectively, compared to the control group. These values were significantly greater than those in both the control and other treatment groups, indicating an optimal promotive effect at 100.0 mg/L. Notably, the treatments at 50.0 mg/L and 200.0 mg/L also enhanced growth parameters compared to the control, albeit to a lesser degree than the 100.0 mg/L treatment. This suggests a non-linear dose–response relationship, with 200.0 mg/L potentially approaching a threshold beyond which the growth-promoting effects diminish. These findings underscore the potential of welan gum as a biostimulant in agriculture, particularly at an optimal concentration of 100.0 mg/L.

### 2.3. Welan Gum Regulates Nitrogen Use Efficiency (NUE) in Maize Seedlings

**Figure 3:**
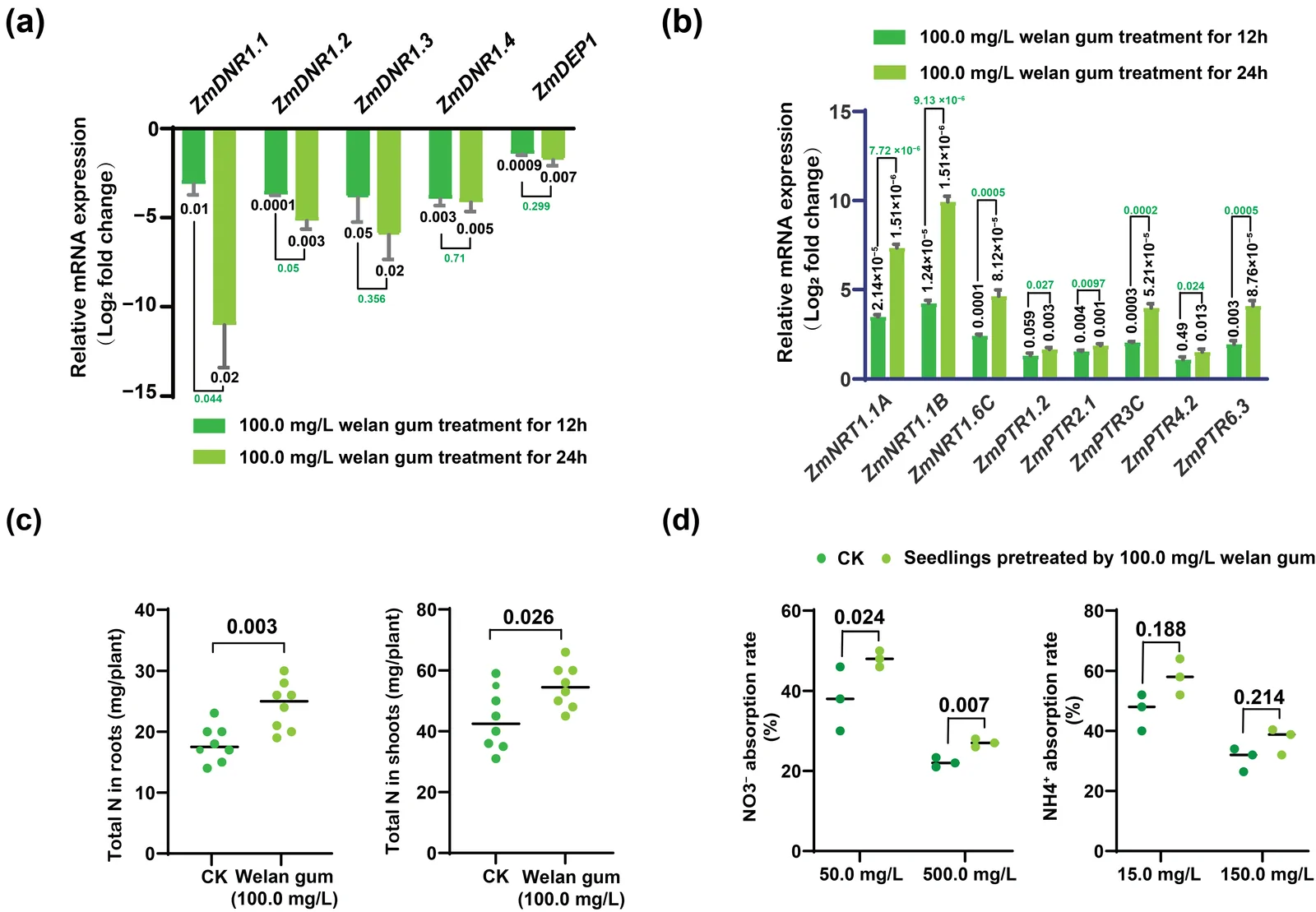
Welan gum improves nitrogen use efficiency (NUE) in maize seedlings. (**a**) Relative expression of *ZmDNR1.1–1.4* and *ZmDEP1* in maize roots treated with 100.0 mg/L welan gum for 12 and 24 h, analyzed by real-time PCR. (**b**) Relative expression of nitrate transporter genes (*ZmNRT1.1A*, *ZmNRT1.1B*, *ZmNRT1.6C*, *ZmPTR1.2*, *ZmPTR2.1*, *ZmPTR3C*, *ZmPTR4.2*, and *ZmPTR6.3*) under the same treatment conditions. For (**a**,**b**), expression levels were normalized to *ZmUBQ* and are shown relative to untreated roots. Error bars represent standard error (*n* = 3). *p* values displayed on the graph are color-coded: black *p* values indicate statistical differences between the treatment and the control (CK) at each time point, while green *p* values indicate statistical differences between the 12 h and 24 h time points under the same treatment. (**c**) Nitrogen content in roots and shoots of control (CK) and welan gum-treated plants. Individual data points from 8 biological replicates per treatment are shown as scatter plots, with *p* values indicated. (**d**) Ionic nitrogen uptake dynamics of maize roots. Seedlings were pretreated with 100.0 mg/L welan gum for 24 h, then exposed to different nitrogen sources: 50.0 and 500.0 mg/L KNO_3_, or 15.0 and 150.0 mg/L NH_4_Cl. NO_3_^−^ and NH_4_^+^ uptake rates are presented as scatter plots, where each point represents pooled measurements from ten seedlings. *p* values are displayed on the graph.

High-molecular-weight EPS have been regarded primarily as agents that improve soil structure, facilitate nutrient uptake, or confer stress protection [15,16]. A previous study in rice showed that welan gum could improve NUE [18], highlighting its potential to reduce nitrogen fertilizer application. To investigate whether this NUE-enhancing effect is broadly conserved across different crop species and to elucidate the underlying mechanisms, we analyzed the expression of key nitrogen utilization genes in maize. Compared to the untreated control, welan gum treatment significantly downregulated the expression of negative regulators of nitrogen responses, including *ZmDNR1.1–1.4* and *ZmDEP1* (Figure 3a). Notably, the suppression of both *ZmDNR1.1* and *ZmDNR1.2* was significantly more pronounced at 24 h than at 12 h. Concurrently, relative to the control, welan gum treatment significantly upregulated multiple transporter genes crucial for nitrogen uptake, translocation, and mobilization (Figure 3b), including the high-affinity nitrate transporters *ZmNRT1.1A*, *ZmNRT1.1B*, and *ZmNRT1.6C*, as well as the peptide transporters *ZmPTR1.2*, *ZmPTR2.1*, *ZmPTR3C*, *ZmPTR4.2*, and *ZmPTR6.3*. In particular, the transcript levels of these transporter genes were significantly higher at 24 h than at 12 h, indicating a sustained and escalating activation of nitrogen transport. Collectively, these temporal dynamics reveal that welan gum treatment triggers a time-dependent, coordinated transcriptional reprogramming that represses negative regulators and promotes active transport, thereby enhancing nitrogen mobilization capacity in maize. Furthermore, treated seedlings showed enhanced nitrogen allocation, with 34.7% and 24.8% higher accumulation in roots and shoots, respectively, compared to levels in the control plants (Figure 3c). Additionally, there was a significant increase in nitrogen uptake efficiency in plants with NO_3_^−^ absorption improving by 26.3% with 50.0 mg/L KNO_3_ treatment and by 22.0% with 500.0 mg/L KNO_3_ treatment compared to the control plants. Similarly, NH_4_^+^ assimilation rose by 24.2% with 15.0 mg/L NH_4_Cl treatment and by 20.3% with 150.0 mg/L NH_4_Cl treatment in comparison to the control plants (Figure 3d). These findings indicate that welan gum enhances nitrogen utilization through coordinated transcriptional regulation of nitrogen regulators and transporters, which contributes to increased nitrogen accumulation in tissues and elevated nitrogen uptake rates, thereby promoting maize seedling growth while reducing fertilizer requirements. Collectively, these results suggest that the welan gum not only establishes a favorable physical niche in the rhizosphere but also actively participates in nitrogen uptake and assimilation, highlighting its dual role as a sustainable tool for cereal crop production [35,36].

### 2.4. Welan Gum Triggers TOR Signaling Pathway for Regulating Maize Growth

**Figure 4:**
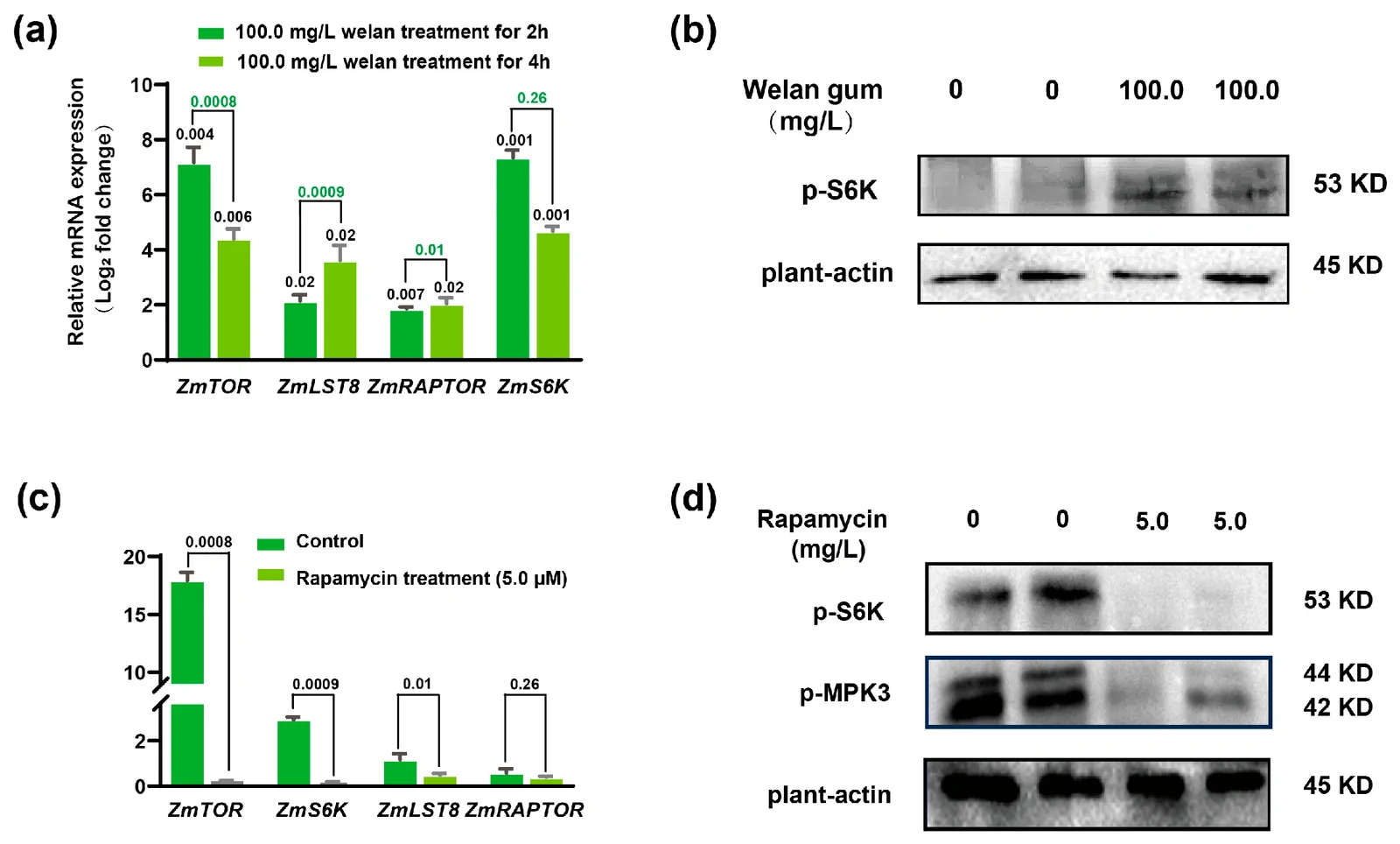
Involvement of the TOR signaling pathway in welan gum-induced maize growth. (**a**) Relative transcript levels of key TOR pathway genes in maize roots treated with welan gum for 2 or 4 h. Expression levels were normalized to *ZmUBQ* and are shown relative to untreated roots. Error bars represent standard error from three biological replicates. *p* values displayed on the graph are color-coded: black *p* values indicate statistical differences between the treatment and the control (CK) at each time point, while green *p* values indicate statistical differences between the 12 h and 24 h time points under the same treatment. (**b**) Phosphorylation of S6K in maize roots after 12 h of welan gum treatment. (**c**) Effect of rapamycin on the expression of TOR pathway genes. Seedlings were treated with welan gum in the absence or presence of rapamycin, and transcript levels were determined. Error bars represent standard error (*n* = 3 biological replicates), and *p* values are indicated. (**d**) Effect of rapamycin on welan gum-induced phosphorylation of MPK3 and S6K in maize roots.

Plants perceive rhizosphere microorganisms and their metabolites through microbe-associated molecular patterns (MAMPs), which trigger intracellular signaling cascades [37]. Among these, the TOR kinase, a member of the phosphoinositide 3-kinase-related protein kinase family, acts as a central integrator of sugar, nitrogen, and energy signals to promote growth [38,39]. Unlike other eukaryotes, plants possess a single TOR complex (TORC), composed of the TOR kinase, RAPTOR, and LST8 [40,41]. Recent studies have suggested that polysaccharides might function as signaling molecules via TOR [42,43]. To investigate whether welan gum modulates TOR signaling in maize, we examined the expression of key TOR-related genes using real-time PCR (Figure 4a). Our analysis revealed that, compared to the untreated control, there was a significant upregulation of major TORC components, including *ZmTOR*, *ZmLST8*, and *ZmRAPTOR*, as well as the downstream substrate *ZmS6K*. Regarding their temporal expression, *ZmTOR* peaked at 2 h and significantly declined at 4 h, whereas *ZmLST8* and *ZmRAPTOR* both showed progressive upregulation from 2 h to 4 h. Meanwhile, *ZmS6K* remained highly expressed with no significant difference between the two time points. Additionally, Western blot analysis further confirmed elevated S6K phosphorylation, a canonical readout of TOR activity, thereby demonstrating that welan gum directly activates the TOR pathway (Figure 4b). These results provide evidence linking an exogenous bacterial exopolysaccharide to TOR activation in plants. To further validate the functional requirement of TOR signaling in welan gum-induced growth promotion, we applied rapamycin at 5.0 μM, a concentration previously established to specifically inhibit TOR activity in maize [44,45,46]. Rapamycin treatment suppressed the expression of *ZmTOR*, *ZmLST8*, and *ZmS6K*, as well as ZmS6K phosphorylation, and this suppression was accompanied by reduced maize growth relative to untreated controls after 10 days of welan gum exposure (Figure 4c, Table S6), demonstrating that TOR signaling is required for welan gum-induced growth promotion. Notably, the phosphorylation of ZmMPK3, the terminal MAPK component in the three-tier MAPK cascade, was also markedly decreased (Figure 4d). This finding suggested a potential crosstalk between TOR and MAPK signaling, leading us to investigate the role of MAPKs in welan gum-mediated growth regulation.

### 2.5. ZmMPK3-1 Is Involved in Welan Gum-Induced Regulation of Maize Growth

**Figure 5:**
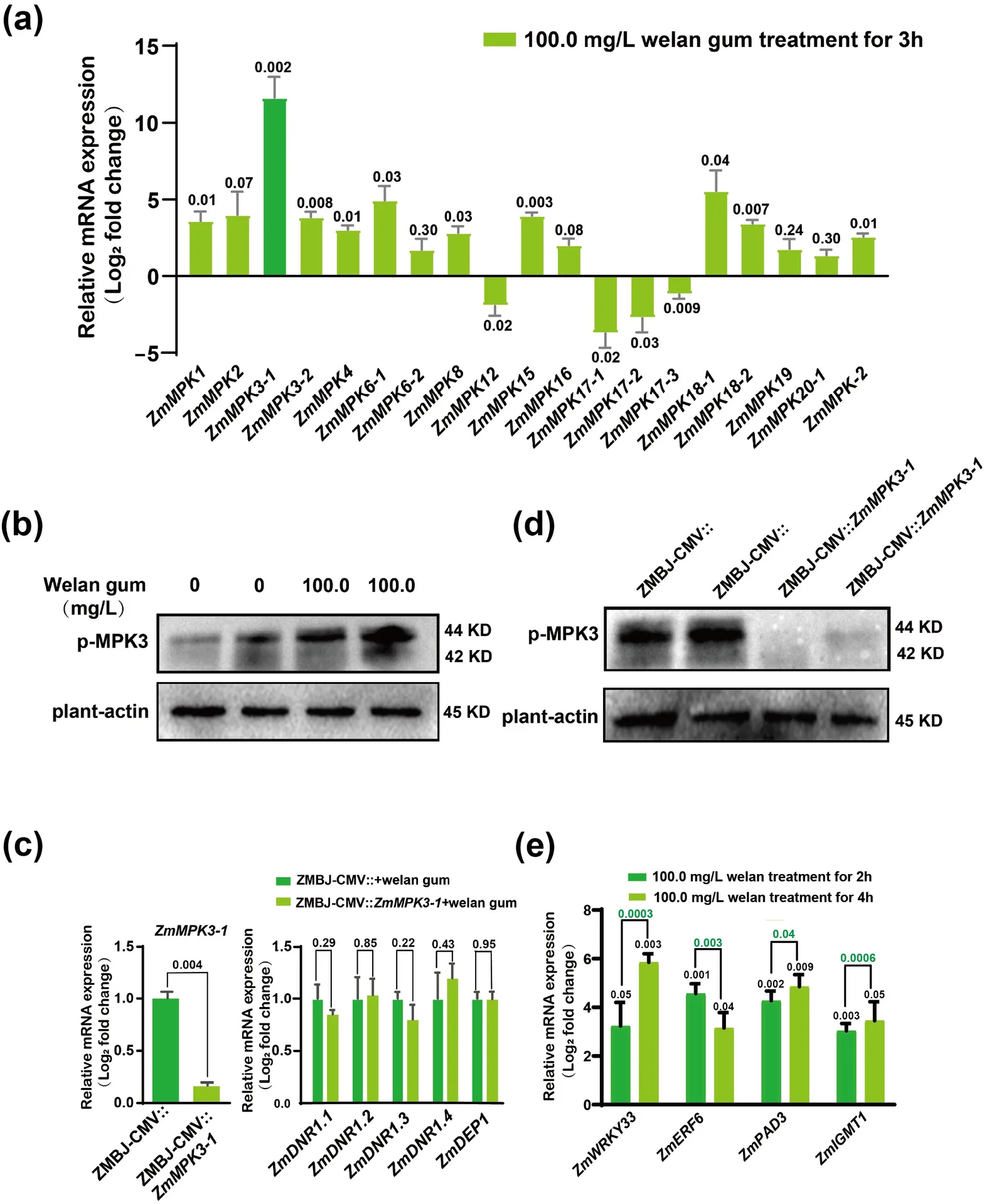
*ZmMPK3-1* mediates welan gum-induced maize growth. (**a**) Relative transcript levels of MPK family members in maize roots treated with welan gum for 3 h. Expression was normalized to *ZmUBQ* and is shown relative to untreated controls. Data are presented as mean ± standard error (*n* = 3 biological replicates), and *p* values are shown on the graph. (**b**) Phosphorylation of MPK3 in maize roots after 16 h of welan gum treatment. (**c**) Relative expression of *ZmMPK3-1* and NUE-related genes in control (empty ZMBJ-CMV vector) and *ZmMPK3-1*-silenced maize roots after welan gum treatment. Seedlings were subjected to VIGS for 2 weeks before the treatment. Expression was normalized to *ZmUBQ* and is shown relative to the empty vector control. Error bars represent standard error (*n* = 3 biological replicates), and *p* values are indicated. (**d**) Phosphorylation of MPK3 in control (empty vector) and *ZmMPK3-1*-silenced maize roots. (**e**) Relative transcript levels of defense genes associated with MPK3 targets in maize roots upon welan gum treatment. Expression was normalized to *ZmUBQ* and is shown relative to untreated controls. Data are presented as mean ± standard error (*n* = 3 biological replicates). *p* values displayed on the graph are color-coded: black *p* values indicate statistical differences between the treatment and the control (CK) at each time point, while green *p* values indicate statistical differences between the 12 h and 24 h time points under the same treatment.

Given the broad roles of MAPKs in plant growth, development, and responses to external stimuli [47], we analyzed the transcriptional response of *MPK* genes to welan gum treatment. The results revealed significant upregulation of 15 *MPK* genes in roots, including *ZmMPK1*, *ZmMPK2*, *ZmMPK3-1*, *ZmMPK3-2*, *ZmMPK4*, *ZmMPK6-1*, *ZmMPK6-2*, *ZmMPK8*, *ZmMPK15*, *ZmMPK16*, *ZmMPK18-1*, *ZmMPK18-2*, *ZmMPK19*, *ZmMPK20-1*, and *ZmMPK20-2*, with expression increases ranging from 1.35- to 11.6-fold relative to untreated control roots (Figure 5a). Conversely, *ZmMPK12*, *ZmMPK17-1*, *ZmMPK17-2*, and *ZmMPK17-3* displayed downregulation ranging from 1.2- to 3.75-fold under the same conditions. *ZmMPK3-1*, whose phosphorylation was significantly reduced upon inhibition of TOR signaling, exhibited the highest level of induced expression (11.6-fold). Western blot analysis further confirmed MPK3 activation in maize roots upon welan gum treatment (Figure 5b). Although ZmMPK3-2 shares high sequence similarity with ZmMPK3-1 and exhibits a comparable induction pattern, it displays a distinct tissue-specific expression profile characterized by low abundance in roots (Figure S2), suggesting the possibility of functional diversification between these two isoforms in maize, which warrants further investigation.

As an ortholog of AtMPK3, ZmMPK3-1 is well documented for mediating plant responses to biotic and abiotic stresses [48]. Its strong responsiveness to both welan gum and TOR inhibition suggested its additional role in welan gum-mediated growth regulation. To further elucidate the regulatory function of ZmMPK3-1, we employed a ZMBJ-CMV VIGS system to silence *ZmMPK3-1*. As shown in Figure 5c, it resulted in an 85% reduction in the expression level of *ZmMPK3-1* in VIGS-treated plants (ZMBJ-CMV::*ZmMPK3-1*) compared to the control plants (ZMBJ-CMV::). In *ZmMPK3-1*-silenced plants, welan gum treatment failed to suppress the expression of *ZmDNR1.1–1.4* and *ZmDEP1*. Instead, their transcript levels remained comparable to those in untreated control plants (Figure 5c and Figure S3), indicating that ZmMPK3-1 is required for welan gum-mediated inhibition of NUE suppressors in maize. Furthermore, Western blot analysis demonstrated that the activation of ZmMPK3 was also sharply downregulated in *ZmMPK3-1* silenced plants (Figure 5d).

Plants inherently face a growth–defense trade-off, in which limited resources must be partitioned between growth and defense [49,50]. Considering that ZmMPK3-1 mediates welan gum-induced NUE in maize, and that MAPK signaling can sense and respond to various external stresses to enhance plant immunity, we further investigated whether treatment with welan gum concurrently affects the plant’s defense response while enhancing NUE. The real-time PCR results revealed that treatment with welan gum led to the upregulation of all four selected disease-resistant genes targeted by MPK3 (Figure 5e). Regarding their temporal expression patterns following treatment, *ZmWRKY33* (WRKY transcription factor 33), *ZmPAD3* (Phytoalexin deficient 3), and *ZmIGMT1* (Indole glucosinolate methyltransferase 1) were progressively upregulated over time relative to the control, showing significantly higher expression levels at 4 h compared to 2 h. In contrast, *ZmERF6* (Ethylene response factor 6) expression peaked at 2 h and then significantly declined by 4 h. These results indicate that welan gum treatment simultaneously induces the expression of defense-related genes, raising the possibility that ZmMPK3-1 may coordinate growth promotion with immune capacity. Moreover, our previous study in *Camptotheca acuminata* has demonstrated that welan gum promotes the accumulation of camptothecin, thereby contributing to the construction of plant defense systems [51]. Collectively, these results indicate that welan gum simultaneously promotes nitrogen assimilation and defense, thereby reconciling the growth–defense trade-off.

### 2.6. ZmMPK3-1 Directly Interacts with ZmTOR in Maize

**Figure 6:**
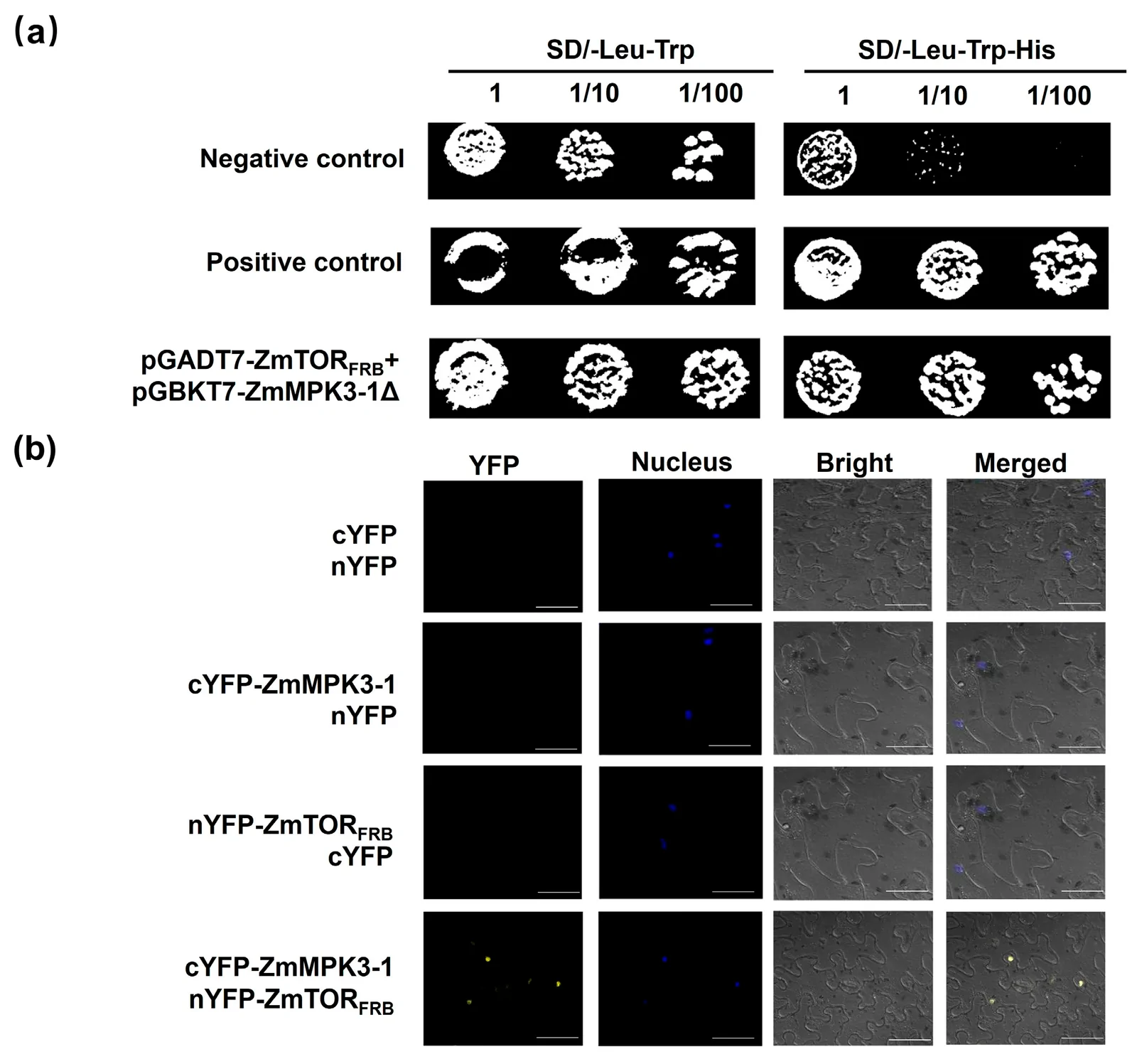
ZmTOR interacts with ZmMPK3-1. Yeast two-hybrid assay for the interaction between ZmTOR_FRB_ and ZmMPK3-1Δ. (**a**) Yeast cells co-expressing the indicated plasmid combinations were spotted on selective medium (SD/-Leu-Trp-His). Growth on this medium indicates a positive interaction. Positive control: pGBKT7-53 + pGADT7-T; negative control: pGBKT7 + pGADT7. (**b**) Bimolecular fluorescence complementation (BiFC) assay in *N. benthamiana* leaf epidermal cells. The N-terminal (nYFP) and C-terminal (cYFP) fragments of YFP were fused to the N-termini of ZmTOR_FRB_ and ZmMPK3-1, respectively, and transiently co-expressed via *Agrobacterium*-mediated transformation. After 72 h, YFP fluorescence was examined by confocal microscopy. Reconstituted YFP signal indicates a direct interaction between ZmTOR and ZmMPK3-1. Scale bar = 250 μm.

Our previous results demonstrated that rapamycin application significantly reduces ZmMPK3 phosphorylation levels, indicating that the root-specific ZmMPK3-1 may participate in TOR signaling-mediated response to welan gum treatment. To characterize the interaction between ZmMPK3-1 and ZmTORC components, we first performed yeast two-hybrid (Y2H) assays. To circumvent potential self-activation, we employed truncated versions consisting of ZmMPK3-1 lacking the kinase domain (ZmMPK3-1Δ) and the FKBP12-rapamycin binding (FRB) domain of ZmTOR (ZmTOR_FRB_). Y2H analysis revealed a direct interaction between ZmMPK3-1Δ and ZmTOR_FRB_ (Figure 6a). Bimolecular fluorescence complementation (BiFC) assays in *N. benthamiana* leaf cells further corroborated these findings, demonstrating strong YFP fluorescence in the nuclei of cells co-expressing cYFP-ZmMPK3-1 and nYFP-ZmTOR_FRB_ (Figure 6b). Together, these assays provide direct evidence of a physical interaction between ZmMPK3-1 and the core TORC component ZmTOR.

### 2.7. ZmLST8 Interacts with and Enhances the Kinase Activity of ZmMPK3-1

**Figure 7:**
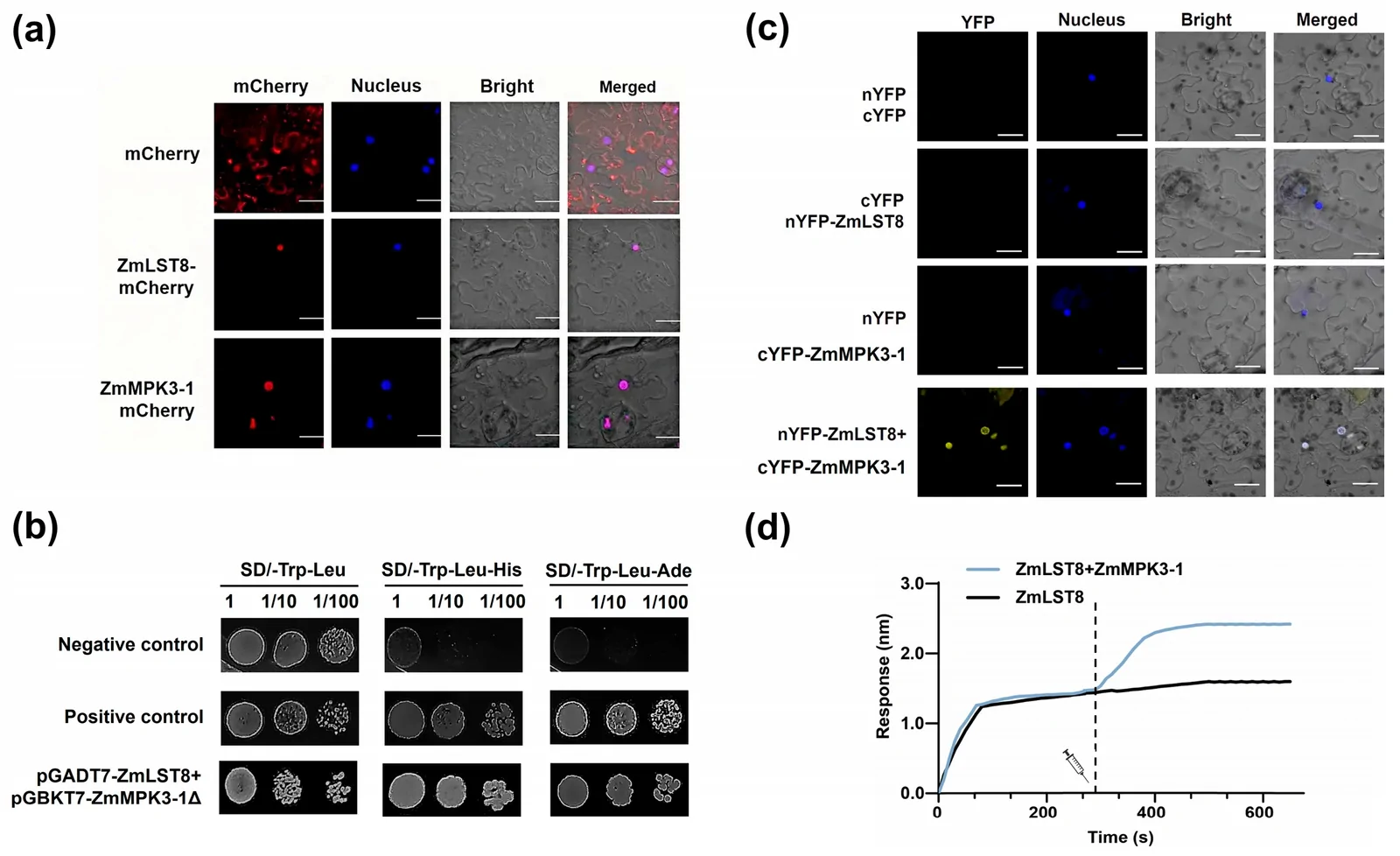
ZmLST8 interacts with ZmMPK3-1. (**a**) Co-localization of ZmLST8 and ZmMPK3-1 in *N. benthamiana* leaf epidermal cells. ZmLST8-mCherry and ZmMPK3-1-mCherry were transiently co-expressed via *Agrobacterium* GV3101. After 48 h, mCherry fluorescence was detected by confocal microscopy; nuclei were stained with DAPI. The mCherry signal overlapped with DAPI staining, indicating that both proteins localize to the nucleus. Scale bar = 250 μm. (**b**) Yeast two-hybrid assay for the interaction between ZmLST8 and ZmMPK3-1. Yeast cells co-expressing the indicated plasmid combinations were spotted on selective medium (SD/-Leu-Trp-His). Growth on this medium indicates a positive interaction. Positive control: pGBKT7-53 + pGADT7-T; negative control: pGBKT7 + pGADT7. (**c**) BiFC assay for the interaction between ZmLST8 and ZmMPK3-1. nYFP was fused to the N-terminus of ZmMPK3-1, and cYFP was fused to the C-terminus of ZmLST8. The resulting constructs were transiently co-expressed in *N. benthamiana* leaf epidermal cells via *Agrobacterium*-mediated transformation. After 72 h, YFP fluorescence was examined by confocal microscopy. Reconstituted YFP signal indicates a direct interaction between ZmLST8 and ZmMPK3-1. Scale bar = 250 μm. (**d**) Bio-layer interferometry (BLI) assay for the interaction between ZmLST8 and ZmMPK3-1. ZmLST8 was first immobilized on the biosensor, and ZmMPK3-1 was then applied as the analyte. Representative binding curves from three independent experiments are shown.

PDBePISA-guided docking further indicated that ZmLST8 exhibits the most favorable binding to ZmMPK3-1 among the TOR components, with larger interface areas and lower estimated binding free energies (Table S7). Both proteins were predicted to localize to the nucleus (Table S8), which we validated by confocal imaging of mCherry-tagged constructs transiently expressed in *N. benthamiana* leaves (Figure 7a). The physical interaction between ZmLST8 and ZmMPK3-1 was verified through multiple complementary approaches. Y2H assays demonstrated direct protein–protein interaction (Figure 7b), while BiFC assays in *N. benthamiana* leaf cells revealed strong nuclear YFP fluorescence upon co-expression of cYFP-ZmMPK3-1 and nYFP-ZmLST8 (Figure 7c). Bio-layer interferometry (BLI) analysis further confirmed specific binding between ZmMPK3-1 and ZmLST8, exhibiting a robust interferometric response during the interaction phase, whereas ZmLST8 alone displayed minimal signal drift (Figure 7d). These results establish that, beyond the direct interaction between ZmTOR and ZmMPK3-1, ZmLST8 also physically associates with ZmMPK3-1, thereby revealing intricate regulatory connections between the TORC and ZmMPK3-1.

**Figure 8:**
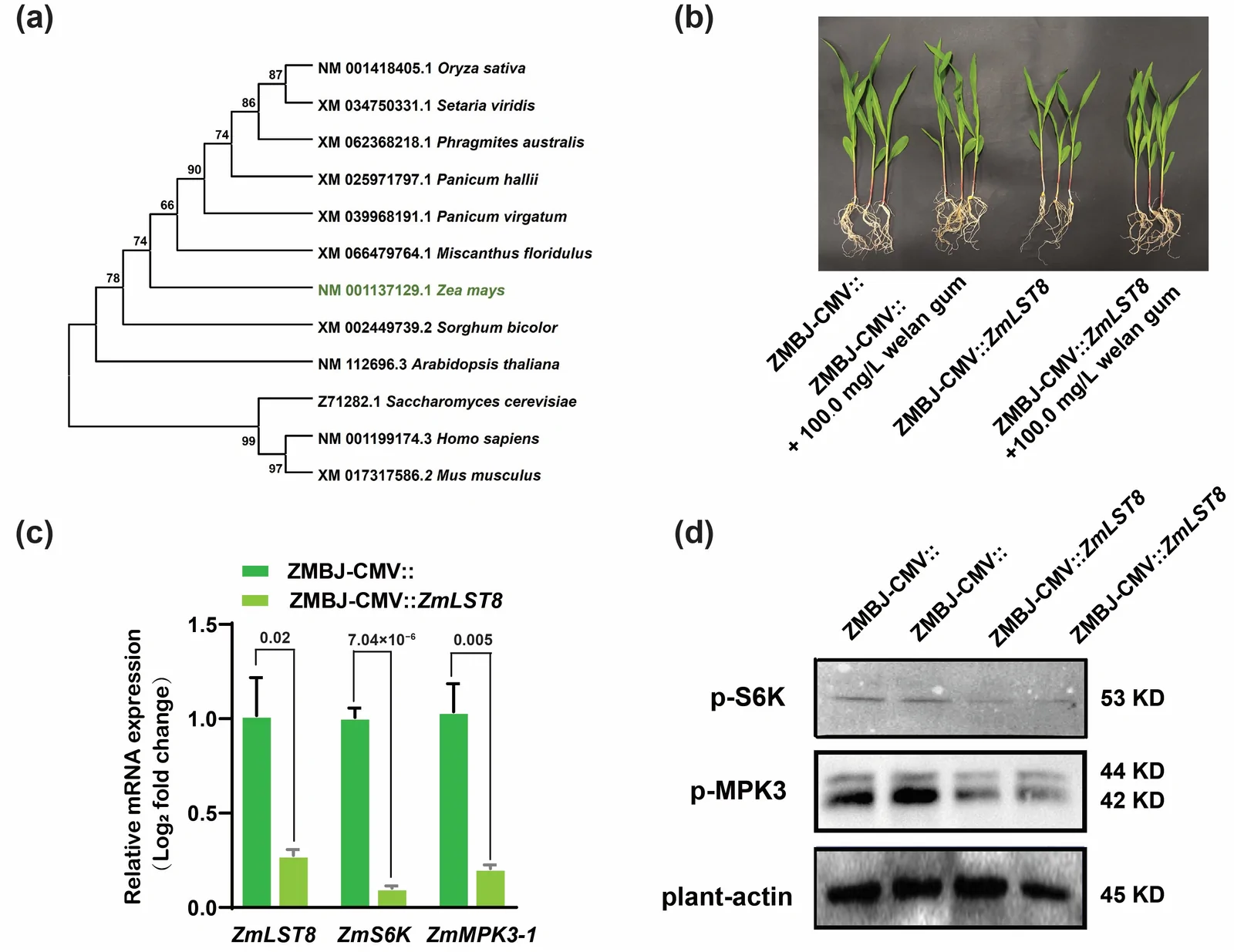
ZmLST8 modulates ZmMPK3-1 transcript abundance, phosphorylation status, and kinase activity. (**a**) Phylogenetic analysis of LST8 proteins from maize (ZmLST8) and 11 other species. The tree was constructed using the maximum likelihood method in MEGA 11.0, based on protein sequences aligned with MUSCLE. Bootstrap values (1000 replicates) are shown at the nodes. (**b**) Phenotypic effect of *ZmLST8* silencing in maize seedlings. Control (empty ZMBJ-CMV vector) and *ZmLST8*-silenced seedlings were treated with 100.0 mg/L welan gum. (**c**) Relative transcript levels of *ZmLST8*, *ZmS6K*, and *ZmMPK3-1* in maize roots 2 weeks after VIGS treatment. mRNA abundance was quantified by real-time PCR, normalized to *ZmUBQ*, and expressed relative to the empty vector control. Error bars indicate standard errors from three biological replicates and *p* values are shown on the graph. (**d**) Western blot analysis of ZmS6K and ZmMPK3 phosphorylation in control and *ZmLST8*-silenced maize roots after welan gum treatment.

In plants, LST8 generally acts as a stabilizing cofactor of the plant TOR complex, promoting efficient and selective phosphorylation of recruited substrates by maintaining the active conformation and integrity of the catalytic core. Phylogenetic analysis demonstrated high conservation of LST8 across cereal species (Figure 8a, Figure S4), and previous research has established a correlation between LST8 absence and inhibited growth [52]. Utilizing the ZMBJ-CMV VIGS system to silence *ZmLST8* expression, we observed significant impairment in the growth of maize seedlings, consistent with the developmental defects reported in other species. This growth inhibition was evident when compared to control seedlings, irrespective of welan gum treatment (Figure 8b, Table S9). Real-time PCR revealed that *ZmLST8* silencing was associated with reduced expression of *ZmS6K* and *ZmMPK3-1* (Figure 8c). Furthermore, Western blot analysis demonstrated diminished MPK3 phosphorylation in *ZmLST8*-silenced maize plants treated with welan gum, indicating that ZmLST8 is required for maintaining both transcript abundance and phosphorylation status of MPK3 under welan gum treatment (Figure 8d).

**Figure 9:**
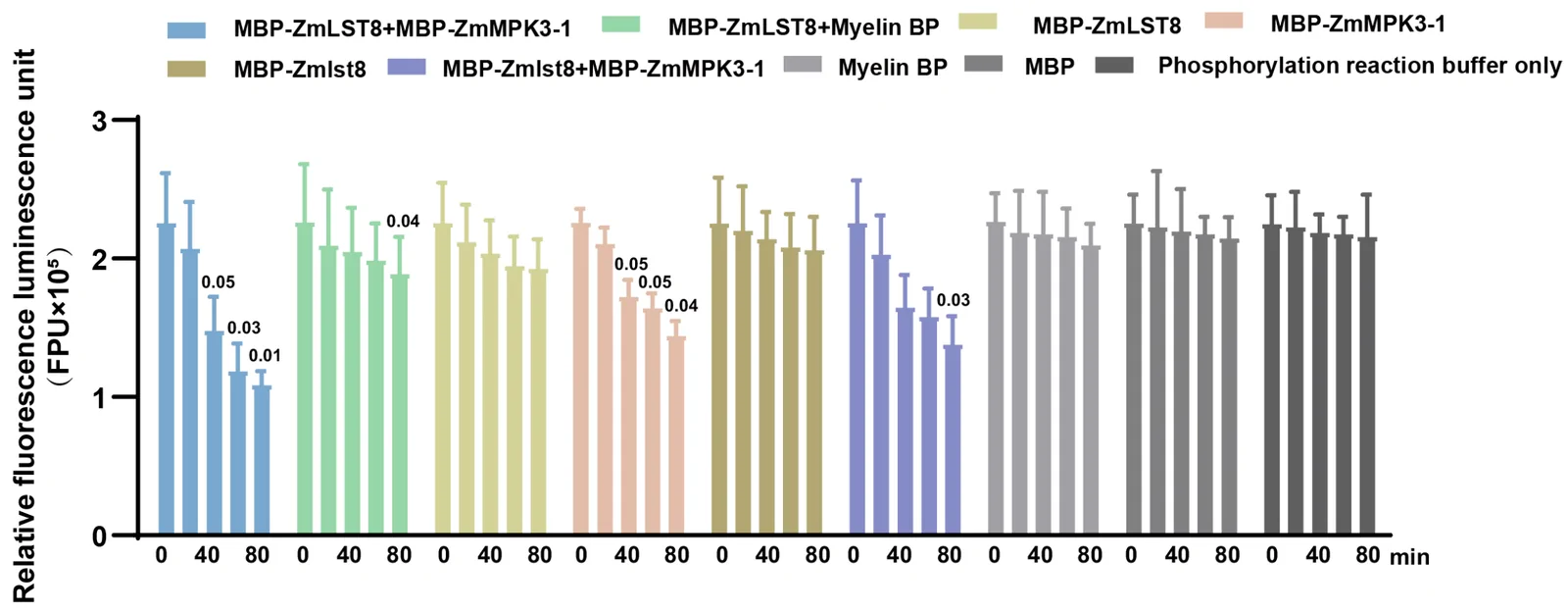
ZmLST8 independently enhances phosphorylation activity of ZmMPK3-1. Kinase activity was monitored by continuous fluorescence recording for 80 min. MBP-ZmMPK3 was used as substrates, and MBP-ZmLST8 was incubated alone or with the substrates as the experimental groups. MBP and MBP-Zmlst8 (boiling-inactivated ZmLST8) incubated alone or with substrates were used as the negative control. Error bars represent standard errors from three biological replicates and *p* values are shown on the graph in comparing with each initial fluorescence luminescence.

Subsequently, we evaluated the biological consequence of this interaction by measuring ZmMPK3-1 kinase activity in an 80-min continuous assay. The fluorescence value ratio, reflecting ZmMPK3-1 activity, gradually declined in both the “maltose-binding protein (MBP)-ZmMPK3” and “MBP-ZmLST8 + MBP-ZmMPK3” groups, suggesting ongoing phosphorylation and ATP depletion (Figure 9). Meanwhile, analysis of the fluorescence decay rate revealed that the combination of MBP-ZmLST8 and MBP-ZmMPK3 exhibited a significantly higher rate than MBP-ZmMPK3 alone, despite ZmLST8 lacking intrinsic phosphorylation activity. These findings suggest that the interaction between ZmLST8 and ZmMPK3-1 substantially enhances the kinase activity of ZmMPK3-1. LST8 was initially identified as a mutation causing secretory pathway defects in yeast [52], and it is now recognized as a core scaffolding component of TORC, with its tryptophan aspartic acid 40 (WD40) repeat domain accommodating multiple partners for signal transduction [53,54,55,56,57,58]. Whereas LST8 is conventionally recognized for stabilizing the TOR kinase, our findings suggest its functions extend beyond this structural role, indicating that LST8 acts as a direct molecular link that regulates the terminal MAPK cascade, independent of its role in stabilizing the TOR kinase. In contrast to the traditional view that TOR and MAPK pathways often act antagonistically in plants [59,60], our study identifies a coordinated regulatory mechanism in which TOR signaling positively influences ZmMPK3-1 activity upon welan gum treatment, thereby reconciling the growth–defense trade-off.

## 3. Conclusions

**Figure 10:**
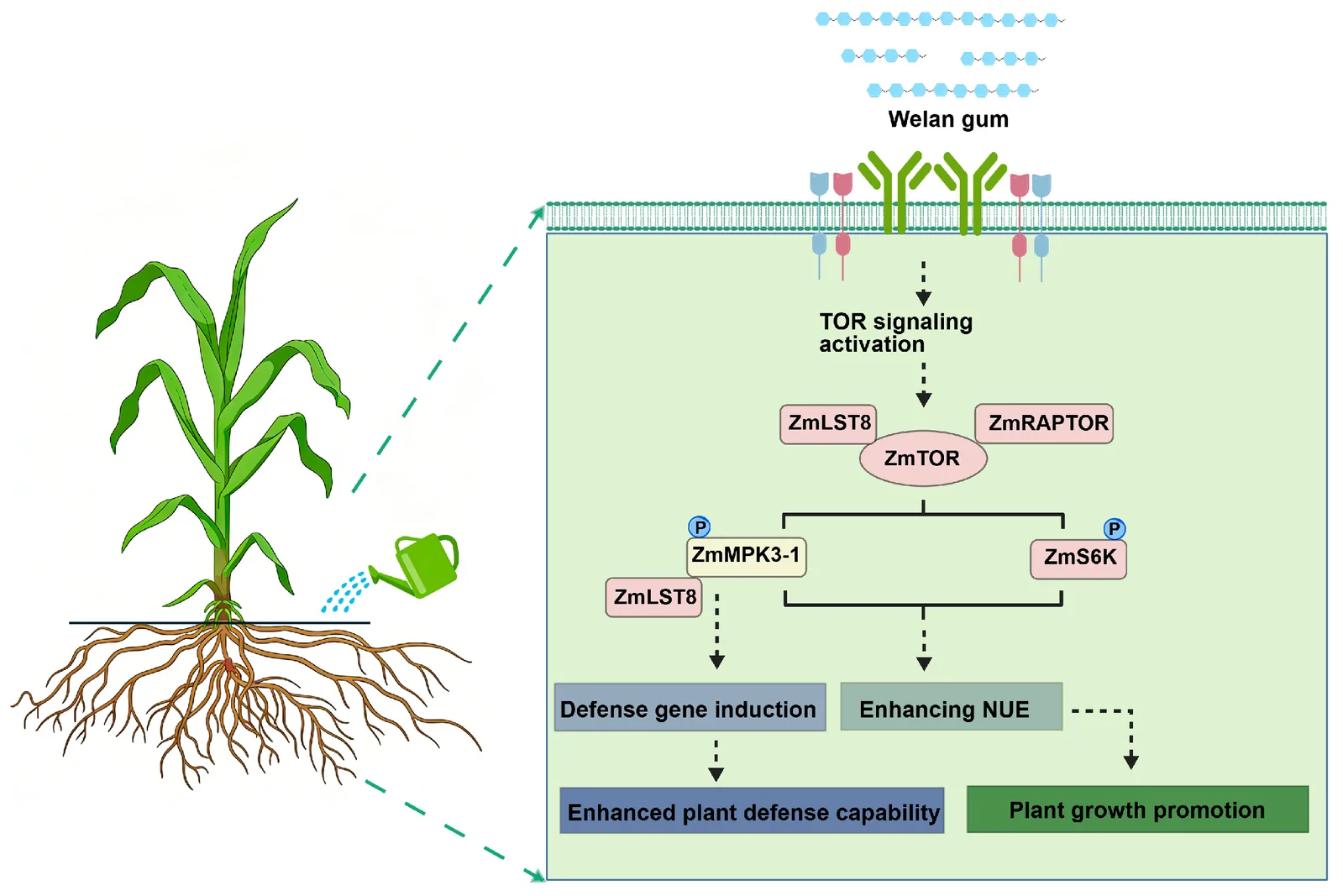
Schematic diagram of welan gum-induced maize growth via TOR and MAPK signaling.

Welan gum, a high-molecular-weight exopolysaccharide derived from *Sphingomonas* sp. Y503, significantly enhances NUE and promotes overall growth in maize seedlings due to its excellent physicochemical and rheological properties. Mechanistically, this efficacy is driven by the synergistic activation and crosstalk of the TOR and MAPK signaling pathways. Specifically, the core TOR complex components, ZmTOR and ZmLST8, physically interact with the terminal MAPK ZmMPK3-1, with ZmLST8 independently enhancing MPK3-1 kinase activity. This molecular interplay orchestrates comprehensive transcriptional reprogramming that suppresses negative NUE regulators, upregulates nitrogen transporters, and concurrently induces defense-related genes, successfully reconciling the plant’s growth–defense trade-off (Figure 10). Ultimately, these findings establish welan gum as a highly promising microbial-derived functional biomaterial with strong potential for developing sustainable, gel-based biofertilizers that improve nutrient delivery, elevate crop performance, and reduce reliance on conventional chemical inputs.

## 4. Materials and Methods

### 4.1. Production and Purification of Welan Gum

*Sphingomonas* sp. Y503, originally isolated from a *Catharanthus roseus* field soil (Dalian, China), was cultured in a two-stage process: seed culture in medium A (glucose, beef extract, NaCl) for 24 h, followed by fermentation in medium B (molasses, beef extract, K_2_HPO_4_, KH_2_PO_4_, MgSO_4_) at 30 °C and 200 rpm for 72 h. Biomass was monitored by OD_600_. The exopolysaccharide was precipitated from the cell-free supernatant with ethanol, and the crude product was further purified by re-dissolution, sonication, ethanol precipitation, isopropanol/acetone washing, and vacuum freeze-drying. Yield was expressed as dry weight of purified welan gum per liter of fermentation broth.

### 4.2. Chemical Composition and Purity Analysis

Total sugars were quantified by the phenol–sulfuric acid method (490 nm, glucose standard), residual protein by BCA assay (562 nm, BSA standard), and nucleic acids by UV absorbance at 260 nm. Elemental (C, H, N, S) contents were determined using an EA300 organic elemental analyzer via high-temperature combustion and thermal conductivity detection.

### 4.3. Structural and Rheological Characterization

FTIR spectra were recorded from 4000 to 400 cm^−1^ using KBr pellets (4 cm^−1^ resolution, 32 scans). Weight-average molecular weight (M_w_) and polydispersity (PDI) were obtained by GPC (Agilent 1260, Agilent Technologies, Santa Clara, CA, USA; TSKgel GMPWXL columns; RI detector) with 0.1 M NaNO_3_ eluent at 0.6 mL/min and 35 °C, calibrated against dextran standards. ^1^H NMR spectra were acquired at 600 MHz and 333 K in D_2_O after two D_2_O lyophilization cycles. Rheological measurements were conducted on an MCR 302 rheometer with 50 mm parallel plates using 0.5% (*w*/*v*) welan gum in deionized water (25 °C, mineral oil seal). Steady-shear viscosity was measured from 0.001 to 2000 s^−1^; linear viscoelastic regions were identified by strain sweeps (0.01–100% strain, 10 rad/s); frequency sweeps (0.01–100 rad/s, 1% strain) and temperature sweeps (5–105 °C, 10 rad/s, 1% strain) were performed to obtain mechanical spectra and thermal stability.

### 4.4. Maize Cultivation and Welan Gum Treatment

The hybrid maize cultivar “Dongdan 1331” (*Zea mays* L.), which is widely planted in the main maize-producing areas of China, was chosen for its agronomic importance, reliable response to microbial biostimulants, and genetic uniformity. Seeds were surface-disinfected by successive immersion in 75% (*v*/*v*) ethanol and 2% (*v*/*v*) sodium hypochlorite, and then rinsed four to five times with sterile water. After sterilization, the seeds were sown in pots (21 cm diameter × 17 cm height) filled with 2.0 kg of autoclaved soil and irrigated with deionized water. The soil was a clay loam Alfisol with the following initial properties: pH 6.00, SOC 9.87 g kg^−1^, total N 0.90 g kg^−1^, available P 16.30 mg kg^−1^, and available K 109.90 mg kg^−1^.

Seedlings were grown in a greenhouse under a 16 h light (26 °C)/8 h dark (18 °C) photoperiod. To ensure consistent hydrologic conditions, each pot was irrigated with 500 mL of deionized water every 2 d, maintaining soil moisture at approximately 70% of field capacity. Prior to experimentation, plants were well-watered, and uniform seedlings at the sixth-leaf stage (V6, approximately 30 days after sowing) were selected for treatments. The experiment consisted of a control (CK) with normal irrigation and three treatment groups receiving a single root irrigation with welan gum solutions at concentrations of 50.0, 100.0, and 200.0 mg/L, respectively. All groups were assessed over a 10-day period.

### 4.5. Determination of Plant Growth and Nitrogen (N) Content

Following a 10-day exposure to the welan gum solution, the maize seedlings were harvested. The root systems were gently detached from the soil and rinsed with deionized water until all adhering soil particles were removed. Vegetative growth parameters were then recorded, including shoot height, primary root length, stem diameter, leaf area, and plant fresh weight.

Total nitrogen (N) content was determined by digesting dried plant samples with 98% H_2_SO_4_ and 30% H_2_O_2_, followed by analysis using the Kjeldahl method with a Kjeite™ 9 Analyzer [61]. Total shoot and root nitrogen (mg/plant) were calculated as:

Total N = N concentration (mg/g dry weight) × biomass (g dry weight).

To assess nitrogen uptake, maize seedling roots were first immersed in a 100.0 mg/L welan gum solution for 24 h, followed by a 48-h exposure to modified culture media containing varying nitrogen sources: nitrate (KNO_3_ at 50.0 mg/L and 500.0 mg/L) or ammonium (NH_4_Cl at 15.0 mg/L and 150.0 mg/L). Root nitrogen uptake rate was calculated as:

N uptake rate = (Initial [NO_3_^−^ or NH_4_^+^] − Residual [NO_3_^−^ or NH_4_^+^])/Total root length.

### 4.6. Gene Expression Analysis

To investigate the effects of welan gum on the TOR and MAPK signaling pathways, nitrogen translocation, and the expression of genes related to NUE in maize roots, total RNA was isolated from each treatment using TRIzol reagent according to the manufacturer’s instructions. The RNA was then reverse-transcribed into cDNA with a commercial reverse transcription kit to prepare templates for subsequent real-time PCR. Each qPCR assay was carried out in a 20-µL reaction mixture comprising SYBR Green Supermix, 0.2 µM of each primer, and an amount of cDNA corresponding to 5 ng of total RNA. The primer details are given in Table S2. The thermal cycling program consisted of an initial denaturation at 95 °C for 1 min, followed by 40 cycles of 95 °C for 20 s, 50 °C for 15 s, and 72 °C for 15 s. Relative transcript levels were determined by the 2^−ΔΔCt^ method, with normalization against the reference gene *ZmUBQ* [62].

### 4.7. Western Blot Analysis

Total protein was extracted from maize roots by homogenizing the tissues in RIPA buffer containing 2% protease inhibitor cocktail (Promega, Madison, WI, USA). The protein concentration was measured with a BCA Protein Assay Kit (Beyotime Biotech, Shanghai, China). Aliquots of 40–60 µg protein per sample were resolved by SDS-PAGE and subsequently transferred onto PVDF membranes. The membranes were blocked with 5% non-fat milk at room temperature for 60 min, and then incubated overnight at 4 °C with primary antibodies recognizing Phospho-p44/42 ERK1/2 (Thr202/Tyr204, #4370T; targets the conserved T-E-Y motif), Phospho-S6K (Thr499, #ab207399), and Plant actin (bsm-33128, used as a loading control). After incubation with the corresponding secondary antibodies for 1 h at room temperature, protein bands were detected by chemiluminescence using ECL Select Western Blot Detection Reagent (Millipore, Burlington, MA, USA) and imaged with a Tanon 5500 system. Each experiment was performed at least twice with consistent results.

### 4.8. Subcellular Localization Analysis

The putative subcellular localization of representative components in the TOR and MAPK pathways was first predicted in silico using DeepLoc 2.0 with default parameters. For experimental validation, the coding sequences of ZmLST8 and ZmMPK3-1 were separately fused with mCherry and cloned into the pBI121GD vector under the control of the CaMV *35S* promoter. The fusion constructs were introduced into *Agrobacterium tumefaciens* GV3101. *Agrobacterium* cultures were grown overnight, collected by centrifugation, washed, and resuspended in infiltration buffer (10 mM MES, 200 μM acetosyringone, 10 mM MgCl_2_) to an OD_600_ of 0.6–0.8. After incubation at 28 °C with shaking for 3 h, the bacterial suspensions were infiltrated into leaves of 4- to 5-week-old *Nicotiana benthamiana* plants (grown at 22–24 °C) using a 1-mL needleless syringe. Fluorescence signals were imaged 48 h post-infiltration under a confocal microscope, with mCherry excited at 580 nm and its emission captured at 610 nm.

### 4.9. Virus-Induced Gene Silencing (VIGS) Assay

CMV-based VIGS assays were performed as previously described [63]. Briefly, 209-bp and 235-bp fragments targeting *ZmMPK3-1* and *ZmLST8*, respectively, were amplified using specific primers (Table S3) and cloned into the pCMV201-2bN81 vector at XbaI/KpnI sites, generating pCMV201-2bN81-*ZmMPK3-1* and pCMV201-2bN81-*ZmLST8* constructs.

Prior to infiltration, *Agrobacterium rhizogenes* C58C1 cultures (OD_600_ = 0.6–0.8) harboring pCMV101, pCMV301, and either pCMV201-2bN81-*ZmMPK3-1* or pCMV201-2bN81-*ZmLST8* were mixed in equal volumes. *A. rhizogenes* C58C1 carrying pCMV201-2bN81-*ZmISPH* was used as a visible control. These suspensions were infiltrated into *N. benthamiana* leaves, which were harvested 3–5 days later to serve as virus sources for maize seed inoculation.

Maize plants subjected to VIGS treatment were hydroponically grown in a controlled growth chamber using Hoagland’s nutrient solution. They were maintained under a 16-h light/8-h dark photoperiod, with temperatures set at 26 °C during the light period and 18 °C during the dark period, over a two-week cultivation period. Successful silencing of *ZmISPH* was confirmed by the photobleached phenotype in leaves (Figure S1). All plants were then treated with welan gum (100.0 mg/L) for 10 days before sampling. 10 biological replicates were collected for gene expression analysis and morphological measurements. Each replicate originated from an independent VIGS-treated plant at the same developmental stage.

### 4.10. Yeast Two-Hybrid Assay

The coding regions of ZmMPK3-1, ZmLST8, a truncated form of ZmMPK3-1 lacking the self-activation domain (ZmMPK3-1Δ), and the FRB domain of ZmTOR (ZmTOR_FRB_) were individually inserted into the pGBKT7 (bait) and pGADT7 (prey) vectors. The experiments were carried out with the Matchmaker Gold Yeast Two-Hybrid System (Clontech) according to the manufacturer’s protocol. Pairs of the resulting recombinant plasmids were co-introduced into *Saccharomyces cerevisiae* strain AH109. The co-transformants were initially selected on synthetic dropout medium lacking tryptophan and leucine (SD/-Trp/-Leu) and then assessed for growth on SD/-Trp/-Leu/-His plates at 30 °C for 4 days. Co-transformation with pGBKT7-53 and pGADT7-T served as the positive control, whereas the empty pGBKT7 and pGADT7 vectors were used as the negative control. All primers used for constructing the Y2H vectors are listed in Table S4.

### 4.11. BiFC Assay

YFP-based BiFC analysis was performed to investigate the interaction between ZmMPK3-1/ZmTOR and ZmLST8 proteins [64]. The split YFP fragments, consisting of the N-terminal (nYFP) and C-terminal (cYFP) portions, were cloned into the pCAMBIA1300 plasmid to generate the nYFP and cYFP expression vectors. *ZmLST8* and *ZmTOR_FRB_* were then fused to the C-terminus of the nYFP fragment, creating the nYFP-*ZmLST8* and nYFP-*ZmTOR_FRB_* fusion constructs, respectively. Similarly, *ZmMPK3-1* were fused to the C-terminus of the cYFP fragment, generating the cYFP-*ZmMPK3-1* fusion constructs. Primers used for cloning are listed in Table S5. The relevant constructs were transformed into *Agrobacterium tumefaciens* GV3101 competent cells and co-infiltrated into *N. benthamiana* epidermal cells. After 72 h of incubation, the reconstitution of the YFP signal was observed under a confocal microscope (ZEISS, Oberkochen, Germany, LSM880) with excitation at 488 nm. Controls groups (nYFP + cYFP, cYFP + nYFP-ZmLST8, cYFP + nYFP-*ZmTOR_FRB_*, nYFP + cYFP-ZmMPK3-1, respectively) were included to ensure the specificity of the assay.

### 4.12. Recombinant Protein Expression, Purification, and Kinase Activity Assay

The *ZmMPK3-1* and *ZmLST8* genes were cloned into the pMAL-c5X vector to generate MBP fusion proteins and expressed in *Escherichia coli* Rosetta (DE3) cells. Recombinant proteins were purified using Amylose Resin according to the manufacturer’s protocol. An inactive form of ZmLST8 (ZmLst8) was produced by boiling the protein at 100 °C for 5 min.

Kinase activity was assessed using the Promega Kinase-Glo® Luminescent Kinase Assay Platform (Cat. No. V3771; Promega, Madison, WI, USA), following the nine experimental group setups described in Cao’s method [65]: One test group (MBP-ZmLST8 + MBP-ZmMPK3-1), one positive control (MBP-ZmMPK3-1), and seven negative controls (MBP, MBP-ZmLST8, MBP-Zmlst8, MBP-Zmlst8 + MBP-ZmMPK3-1, Myelin Basic Protein, MBP-ZmLST8 + Myelin Basic Protein, and buffer only). Phosphorylation reactions (25.0 mM Tris-HCl, pH 7.4; 10.0 mM CaCl_2_; 100.0 mM MgCl_2_; 100.0 µM ATP; 1.0 mM DTT; 1.0 mM PMSF) were incubated at 30 °C, and luminescence was continuously recorded over 80 min in a 60 µL reaction volume.

### 4.13. Bio-Layer Interferometry (BLI) Binding Assay

The interaction between recombinant ZmLST8 and ZmMPK3-1 was analyzed using the Octet^®^ R8 system (Sartorius, Göttingen, Germany) as Hajek’s method [66]. Anti-polyhistidine APS sensors were pre-equilibrated in phosphate-buffered saline (PBS, pH 7.4) for 60 s, then loaded with MBP-tagged ZmLST8. A stable baseline was established by immersing the sensors in PBS for 60–120 s. For binding analysis, sensors were exposed to varying concentrations of ZmMPK3-1 (0–1000 nM in PBS) during a 300-s association phase, followed by a 300-s dissociation phase in PBS to assess complex stability. Parallel sensors immersed in PBS alone served as negative controls to account for nonspecific binding. Data were processed using Octet BLI Analysis Software v12.2. Raw signals were normalized against reference sensors (PBS only), and binding curves were fitted to a 1:1 Langmuir binding model to determine kinetic parameters and equilibrium dissociation constants.

### 4.14. Statistical Analysis

Data were analyzed using SPSS 20.0 software with one-way ANOVA. Statistical significance was assessed by Duncan’s multiple range test at *p* < 0.05.

## Figures and Tables

**Table 1:** Effects of different concentrations of welan gum on vegetative growth parameters of maize seedlings.

Parameters	Control	50.0 mg/L	100.0 mg/L	200.0 mg/L
Height (cm)	40.68 ± 1.65 ^a^	44.42 ± 0.92 ^b^	50.83 ± 1.08 ^c^	43.7 ± 1.32 ^b^
Shoot weight (FW/g)	10.15 ± 1.42 ^a^	13.07 ± 1.76 ^b^	15.43 ± 1.44 ^c^	12.84 ± 1.87 ^b^
Root weight (FW/g)	13.17 ± 2.23 ^a^	16.16 ± 2.41 ^b^	19.99 ± 2.81 ^c^	15.34 ± 3.01 ^a^
Shoot weight (DW/g)	2.43 ± 0.18 ^a^	2.66 ± 0.20 ^b^	3.05 ± 0.08 ^b^	2.69 ± 0.56 ^a^
Root weight (DW/g)	1.87 ± 0.17 ^a^	2.02 ± 0.09 ^a^	2.32 ± 0.22 ^b^	2.02 ± 0.14 ^a^
Leaf area (cm^2^)	474.00 ± 30.02 ^a^	541.81 ± 27.33 ^b^	583.78 ± 89.63 ^c^	548.07 ± 90.16 ^b^

Maize seedlings were grown under respective welan gum treatments for 10 days. Data are presented as mean ± standard error (*n* = 10). Different lowercase letters within a row indicate significant differences among treatments according to one-way ANOVA followed by Duncan’s multiple range test (*p* < 0.05).

## Data Availability

All data that support the findings of this study are included in the manuscript or in the Supplementary Materials. Four supplemental figures and nine supplemental tables: Construction of the ZMBJ-CMV VIGS vector and its functional validation through silencing of *ZmISPH* in maize (Figure S1), real-time PCR analysis of *ZmMPK3-1* and *ZmMPK3-2* expression in root, stem and leaf of maize plants (Figure S2), real-time PCR analysis of NUE genes in ZMBJ-CMV-treated maize subjected to welan gum treatment (Figure S3), multiple alignment of LST8 among various cereal plants (Figure S4); Physicochemical properties and molecular parameters of the extracted welan gum (Table S1), list of primers used for real-time PCR analysis, VIGS vector construction, vector construction in the Y2H assay, and vector construction used in the BiFC assay (Tables S2–S5), maize growth attribute under rapamycin treatment (Table S6), PDBePISA interface results between the selected proteins (Table S7), predicted subcellular localization of selected proteins (Table S8), and effects of *ZmLST8*-1 silencing on growth attribute in maize seedlings (Table S9).

## Supplementary Materials

The following supporting information can be downloaded at: https://www.mdpi.com/article/10.3390/gels12080724/s1, Figure S1: Construction of the ZMBJ-CMV VIGS vector and its functional validation through silencing of *ZmISPH* in maize. (a). Schematic diagram of ZMBJ-CMV-based VIGS vectors; (b). Construction of the pCMV201-2bN81 vector; (c). Photobleaching (chlorosis) phenotypes observed in maize leaves following *ZmISPH* silencing; Figure S2: Real-time PCR analysis of *ZmMPK3-1* and *ZmMPK3-2* expression in root, stem and leaf of maize plants. The results were normalized to *ZmUBQ*. Significance of *ZmMPK3-1* and *ZmMPK3-2* expression in different tissues are shown relative to root and stem, respectively. Error bars represent standard errors with three biological replicates and *p* values are shown on the graph; Figure S3: Real-time PCR analysis of *NUE* genes in ZMBJ-CMV-treated maize subjected to welan gum treatment. Real-time PCR results were normalized to *ZmUBQ* and are shown relative to the levels in the empty vector control group. Error bars represent standard errors from three biological replicates and *p* values are shown on the graph; Figure S4: Multiple alignment of LST8 among various cereal plants. ZmLST8, *Zea mays* LST8; OsLST8, *Oryza sativa* LST8; MfLST8, *Miscanthus floridulus* LST8; SvLST8, *Sorghum virgatum* LST8; SbLST8, *Sorghum bicolor* LST8; PhLST8_WAT1_like, *Panicum Hallii* LST8_WAT1_like; PaLST8-like, *Phragmites australis* LST8-like; PvLST8, *Panicum virgatum* LST8; Table S1: Comparative physicochemical properties of welan gum produced by strain Y503 and a commercial reference; Table S2: List of primers used for real-time PCR analysis; Table S3: List of primers used for VIGS vector construction; Table S4: Primer sequences used for vector construction in the Y2H assay; Table S5: Primer sequences for vector construction used in the BiFC assay; Table S6: Maize growth attribute under rapamycin treatment; Table S7: PDBePISA interface results between the selected proteins; Table S8: Predicted Subcellular Localization of Selected Proteins; Table S9: Effects of *ZmLST8-1* silencing on growth attribute in maize seedlings.

## Abbreviations

The following abbreviations are used in this manuscript:

TOR	target of rapamycin
MAPK	mitogen activated protein kinase
PGPR	plant growth-promoting rhizobacteria
NUE	nitrogen use efficiency
EPS	extracellular polymeric substance
TIAs	terpenoid indole alkaloids
PI3K	phosphoinositide 3 kinase
MAPKKK	MAPK kinase kinase
MKK/MEK/MAP2K	MAPK kinase
VIGS	virus induced gene silencing
Y2H	yeast two hybrid
BiFC	bimolecular fluorescence complementation
BLI	bio layer interferometry
YFP	yellow fluorescent protein
TORC	TOR complex
RAPTOR	regulatory associated protein of TOR
LST8	lethal with sec thirteen 8
S6K	S6 kinase
FRB	FKBP12 rapamycin binding
MBP	maltose binding protein
MAMPs	microbe associated molecular patterns
WD40	tryptophan aspartic acid 40

## References

[1] Khan A.L., Waqas M., Kang S.M., Al-Harrasi A., Hussain J., Al-Rawahi A., Al-Khiziri S., Ullah I., Ali L., Jung H.Y. (2014). Bacterial Endophyte *Sphingomonas* sp. LK11 Produces Gibberellins and IAA and Promotes Tomato Plant Growth. J. Microbiol..

[2] Verbon E.H., Liberman L.M. (2016). Beneficial Microbes Affect Endogenous Mechanisms Controlling Root Development. Trends Plant Sci..

[3] Kong Z., Liu H. (2022). Modification of Rhizosphere Microbial Communities: A Possible Mechanism of Plant Growth Promoting Rhizobacteria Enhancing Plant Growth and Fitness. Front. Plant Sci..

[4] Kumari R., Pandey E., Bushra S., Faizan S., Pandey S. (2024). Plant Growth Promoting Rhizobacteria (PGPR) Induced Protection: A Plant Immunity Perspective. Physiol. Plant..

[5] Mmotla K., Sibanyoni N.R., Allie F., Sitole L., Mafuna T., Mashabela M.D., Mhlongo M.I. (2025). Exploring the Intricacies of Plant Growth Promoting Rhizobacteria Interactions: An Omics Review. Ann. Microbiol..

[6] Gao Y., Wang Z., Cheng J., Fu Y., Wang Y., Sun Y., Geng G., Sun Y. (2026). Effects of *Sphingomonads* on Sugar Beet Growth and Rhizosphere Microbiota under Continuous Cropping. Front. Microbiol..

[7] Xue Y., Loranger M.E.W., Jia Y., Andoy N.M., Moeder W., Yoshioka K., Sullan R.M.A. (2025). Single-Cell Force Spectroscopy Uncovers Root Zone- and Bacteria-Specific Interactions. Angew. Chem..

[8] Luo Y., Wang F., Huang Y., Zhou M., Gao J., Yan T., Sheng H., An L., An L. (2019). *Sphingomonas* sp. Cra20 Increases Plant Growth Rate and Alters Rhizosphere Microbial Community Structure of *Arabidopsis thaliana* Under Drought Stress. Front. Microbiol..

[9] Saha I., Datta S., Biswas D. (2020). Exploring the Role of Bacterial Extracellular Polymeric Substances for Sustainable Development in Agriculture. Curr. Microbiol..

[10] Morcillo R.J.L., Manzanera M. (2021). The Effects of Plant-Associated Bacterial Exopolysaccharides on Plant Abiotic Stress Tolerance. Metabolites.

[11] Gao X., Zhu X., Wang X., Liu X., Guo R.T., Luan J., Liu Z., Yu F. (2025). Modulation of Terpenoid Indole Alkaloid Biosynthesis in *Catharanthus roseus* by *Sphingomonas* Sp Y503 via the CrMAPKKK1-CrMAPKK1/CrMAPKK2-CrMPK3 Signaling Cascade. Plant Cell Environ..

[12] White D.C., Sutton S., Ringelberg D.B. (1996). The Genus *Sphingomonas*: Physiology and Ecology. Curr. Opin. Biotechnol..

[13] Asaf S., Numan M., Khan A.L., Al-Harrasi A. (2020). *Sphingomonas*: From Diversity and Genomics to Functional Role in Environmental Remediation and Plant Growth. Crit. Rev. Biotechnol..

[14] Huang H., Lin J., Li S.X. (2022). Biopolymers Produced by *Sphingomonas* Strains and Their Potential Applications in Petroleum Production. Polymers.

[15] Srivastava S., Sharma S. (2022). Insight into Exopolysaccharide-Mediated Stress Tolerance in Plants: A Feasible Approach Towards the Development of Next-Generation Bioformulations. J. Soil Sci. Plant Nutr..

[16] Costa O.Y.A., Raaijmakers J.M., Kuramae E.E. (2018). Microbial Extracellular Polymeric Substances: Ecological Function and Impact on Soil Aggregation. Front. Microbiol..

[17] Luo H., Wang F., Wang L., Li Y., Yang M., Zhang H. (2025). Microbial Welan Gum Production, Chemistry and Applications: A Review. Int. J. Biol. Macromol..

[18] Xu X.Y., Sun L., Li S., Xu H., Lei P. (2020). Welan Gum Promoted the Growth of Rice Seedlings by Enhancing Carbon and Nitrogen Assimilation. Carbohydr. Res..

[19] Jiang C.H., Fan Z.H., Xie P., Guo J.H. (2016). *Bacillus cereus* AR156 Extracellular Polysaccharides Served as a Novel Micro-Associated Molecular Pattern to Induced Systemic Immunity to Pst DC3000 in *Arabidopsis*. Front. Microbiol..

[20] Kumawat K.C., Sharma B., Nagpal S., Kumar A., Tiwari S., Nair R.M. (2023). Plant Growth-Promoting Rhizobacteria: Salt Stress Alleviators to Improve Crop Productivity for Sustainable Agriculture Development. Front. Plant Sci..

[21] Zhao Y., Wang X.Q. (2021). The Hot Issue: TOR Signalling Network in Plants. Funct. Plant Biol..

[22] Sun T., Zhang Y. (2022). MAP Kinase Cascades in Plant Development and Immune Signaling. EMBO Rep..

[23] Manna M., Rengasamy B., Singh A.K. (2023). Revisiting the Role of MAPK Signalling Pathway in Plants and Its Manipulation for Crop Improvement. Plant Cell Environ..

[24] Qadri T.A., Khan A., Khan R.A. (2026). Nanofertilizers for Sustainable Agriculture: Mechanisms, Benefits, and Environmental Challenges. BioNanoScience.

[25] Melara F., da Silva L.K., Martins Sanderi D. (2026). Enhanced Efficiency Fertilizer: A Review on Technologies, Perspectives, and Research Strategies. Environ. Dev. Sustain..

[26] Wang Y., Zhu S., Li Z., Zhang T., Jiang Y., Zhu L., Zhan X. (2024). Glycerol-Driven Adaptive Evolution for the Production of Low-Molecular-Weight Welan Gum: Characterization and Activity Evaluation. Carbohydr. Polym..

[27] Tako M., Sakae A., Nakamura S. (1989). Rheological Properties of Gellan Gum in Aqueous Media. Agric. Biol. Chem..

[28] Lin J.-Y., Deng J., Huang Z., Dong H., Chang A., Zhu H. (2023). Physicochemical and Structural Characterization of Alkali-Treated Biopolymer Sphingan WL Gum from Marine *Sphingomonas* sp. WG. ACS Omega.

[29] Xu L., Xu G., Liu T., Chen Y., Gong H. (2013). The Comparison of Rheological Properties of Aqueous Welan Gum and Xanthan Gum Solutions. Carbohydr. Polym..

[30] Kaur V., Bera M.B., Panesar P.S., Kumar H., Kennedy J.F. (2014). Welan Gum: Microbial Production, Characterization, and Applications. Int. J. Biol. Macromol..

[31] Lei J., Gao J., Lin X., Zhu H., Wang X. (2026). Carboxyl-Grafted Welan Gum for Enhanced Green Corrosion Inhibition Performance in Acidic Environments Under Rising Temperatures. Coatings.

[32] Gantait A., Masih S.A., Maxton A. (2026). Rhizobacteria Exopolysaccharide: A Boon in Reclaiming Soil Fertility, Augmenting Plant Growth and Plant Stress Resilience. Environ. Microbiol. Rep..

[33] Abou-alfitooh S.A.M., El-hoshoudy A.N. (2023). Eco-Friendly Modified Biopolymers for Enhancing Oil Production: A Review. J. Environ. Polym. Degrad..

[34] Dar A., Dar A., Zahir Z.A., Iqbal M., Mehmood A., Javed A., Hussain A., Bushra, Ahmad M. (2021). Efficacy of Rhizobacterial Exopolysaccharides in Improving Plant Growth, Physiology, and Soil Properties. Environ. Monit. Assess..

[35] Liu X., Yao T. (2024). Types, Synthesis Pathways, Purification, Characterization, and Agroecological Physiological Functions of Microbial Exopolysaccharides: A Review. Int. J. Biol. Macromol..

[36] Misra S., Salimi F., Farrokh P. (2026). The Biostimulant Role of Microbial Exopolysaccharides: Mechanisms and Agricultural Applications. World J. Microbiol. Biotechnol..

[37] Trdá L., Boutrot F., Claverie J., Brulé D., Dorey S., Poinssot B. (2015). Perception of Pathogenic or Beneficial Bacteria and Their Evasion of Host Immunity: Pattern Recognition Receptors in the Frontline. Front. Plant Sci..

[38] Caldana C., Martins M.C.M., Mubeen U., Urrea-Castellanos R. (2019). The Magic “hammer” of TOR: The Multiple Faces of a Single Pathway in the Metabolic Regulation of Plant Growth and Development. J. Exp. Bot..

[39] Liu Y., Xiong Y. (2022). Plant Target of Rapamycin Signaling Network: Complexes, Conservations, and Specificities. J. Integr. Plant Biol..

[40] Wang F., Wei Y., Yan T., Wang C., Chao Y., Jia M., An L., Sheng H. (2022). *Sphingomonas* sp. Hbc-6 Alters Physiological Metabolism and Recruits Beneficial Rhizosphere Bacteria to Improve Plant Growth and Drought Tolerance. Front. Plant Sci..

[41] Martínez Pacheco J., Canal M.V., Pereyra C.M., Welchen E., Martínez-Noël G.M.A., Estevez J.M., Estevez J.M. (2021). The Tip of the Iceberg: Emerging Roles of TORC1, and Its Regulatory Functions in Plant Cells. J. Exp. Bot..

[42] Méndez-Gómez M., Castro-Mercado E., Peña-Uribe C.A., Reyes de la Cruz H., López-Bucio J., García-Pineda E. (2020). *Azospirillum brasilense* Sp245 Lipopolysaccharides Induce Target of Rapamycin Signaling and Growth in *Arabidopsis thaliana*. J. Plant Physiol..

[43] Scarpin M.R., Simmons C.H., Brunkard J.O. (2022). Translating across Kingdoms: TOR Promotes Protein Synthesis through Conserved and Divergent Pathways in Plants. J. Exp. Bot..

[44] Xiong Y., Sheen J. (2012). Rapamycin and Glucose-Target of Rapamycin (TOR) Protein Signaling in Plants. J. Biol. Chem..

[45] Montané M.H., Menand B. (2013). ATP-Competitive mTOR Kinase Inhibitors Delay Plant Growth by Triggering Early Differentiation of Meristematic Cells but No Developmental Patterning Change. J. Exp. Bot..

[46] Salazar-Díaz K., Dong Y., Papdi C., Ferruzca-Rubio E.M., Olea-Badillo G., Ryabova L.A., Dinkova T.D. (2021). TOR Senses and Regulates Spermidine Metabolism during Seedling Establishment and Growth in Maize and *Arabidopsis*. iScience.

[47] Suarez Rodriguez M.C., Petersen M., Mundy J. (2010). Mitogen-Activated Protein Kinase Signaling in Plants. Annu. Rev. Plant Biol..

[48] Wang J., Ding H., Zhang A., Ma F., Cao J., Jiang M. (2010). A Novel Mitogen-Activated Protein Kinase Gene in Maize (*Zea mays*), *ZmMPK3*, Is Involved in Response to Diverse Environmental Cues. J. Integr. Plant Biol..

[49] Zeng Z., Shi J., Qiu C., Fan T., Lü J., Abdelnabby H., Wang M. (2024). Nitrogen Input Reduces the Physical Defense of Rice Plant against Planthopper, *Nilaparvata lugens* (Hemiptera: Delphacidae). J. Econ. Entomol..

[50] Ding S., Shao X., Li J., Ahammed G.J., Yao Y., Ding J., Hu Z., Yu J.Q., Shi K. (2021). Nitrogen Forms and Metabolism Affect Plant Defence to Foliar and Root Pathogens in Tomato. Plant Cell Environ..

[51] Yang B., Ma W., Cheng J. (2026). Welan Gum Promotes Camptothecin Production in *Camptotheca* Acuminata by Activating Multiple Signaling Pathways. Biotechnol. Lett..

[52] Moreau M., Azzopardi M., Clément G., Dobrenel T., Marchive C., Renne C., Martin-Magniette M.-L., Taconnat L., Renou J.-P., Robaglia C. (2012). Mutations in the *Arabidopsis* Homolog of LST8/GβL, a Partner of the Target of Rapamycin Kinase, Impair Plant Growth, Flowering, and Metabolic Adaptation to Long Days. Plant Cell.

[53] Maegawa K., Takii R., Ushimaru T., Kozaki A. (2015). Evolutionary Conservation of TORC1 Components, TOR, Raptor, and LST8, between Rice and Yeast. Mol. Genet. Genom..

[54] Couso I., Pérez-Pérez M.E., Ford M.M., Martínez-Force E., Hicks L.M., Umen J.G., Crespo J.L. (2020). Phosphorus Availability Regulates TORC1 Signaling via LST8 in *Chlamydomonas*. Plant Cell.

[55] Forzani C., Duarte G.T., Van Leene J., Clément G., Huguet S., Paysant-Le-Roux C., Mercier R., De Jaeger G., Leprince A.-S., Leprince A.-S. (2019). Mutations of the *AtYAK1* Kinase Suppress TOR Deficiency in *Arabidopsis*. Cell Rep..

[56] Giannattasio S., Liu Z., Thornton J.M., Butow R.A. (2005). Retrograde Response to Mitochondrial Dysfunction Is Separable from TOR1/2 Regulation of Retrograde Gene Expression. J. Biol. Chem..

[57] Yue Z., Lv Z., Shao Y., Zhang W., Zhao X., Guo M., Li C. (2019). Cloning and Characterization of the Target Protein Subunit Lst8 of Rapamycin in *Apostichopus japonicus*. Fish. Shellfish Immunol..

[58] Zhang Z., Shu Y., Li Y., Xie D., Chen G., Ren J., Zhu H., Zhou H. (2024). *NgLst8* Coactivates TOR Signaling to Activate Photosynthetic Growth in *Nannochloropsis gaditana*. Microorganisms.

[59] Yu Y., Yin Y., Jiang Y., Zhao J., Ma L., Li Z., Chen Q., Meng X.N., Fan H. (2025). Unraveling the Role of TARGET OF RAPAMYCIN in the Immune Response of *Cucumis sativus* to *Podosphaera xanthii*. Physiol. Plant..

[60] Xing H., Bai Y., Ding Q., Wang H., Gao G., Hu Z., Yu Y., Fan H., Meng X., Cui N. (2025). Transcriptomic Analysis of Regulating the Growth and Development of Tomato Seedlings by the Crosstalk between JA and TOR Signaling. Plant Cell Rep..

[61] Pace G.M., MacKown C.T., Volk R.J. (1982). Minimizing Nitrate Reduction during Kjeldahl Digestion of Plant Tissue Extracts and Stem Exudates Application to 15N studies. Plant Physiol..

[62] Pfaffl M.W. (2001). A New Mathematical Model for Relative Quantification in Real-Time RT-PCR. Nucleic Acids Res..

[63] Wang R., Yang X., Wang N., Liu X., Nelson R.S., Li W., Fan Z., Zhou T. (2016). An Efficient Virus-Induced Gene Silencing Vector for Maize Functional Genomics Research. Plant J..

[64] Chen H., Zou Y., Shang Y., Lin H., Wang Y., Cai R., Tang X., Zhou J. (2008). Firefly Luciferase Complementation Imaging Assay for Protein-Protein Interactions in Plants. Plant Physiol..

[65] Cao H., Yang Q., Wang T., Du T., Song Z., Dong B., Chen T., Wei Y., Xue J., Meng D. (2023). Melatonin-Mediated CcARP1 Alters F-Actin Dynamics by Phosphorylation of CcADF9 to Balance Root Growth and Salt Tolerance in Pigeon Pea. Plant Biotechnol. J..

[66] Hajek P., Bader A., Helmstetter F., Henke B., Arnold P., Beitz E. (2019). Cell-Free and Yeast-Based Production of the Malarial Lactate Transporter, PfFNT, Delivers Comparable Yield and Protein Quality. Front. Pharmacol..

