# Supplementary material for: Welan Gum, a Bacterial Polysaccharide, Promotes Maize Seedling Growth Through TOR and MAPK Signaling Pathways
Source: Gels. 2026 Aug 14;12(8):724. doi: 10.3390/gels12080724 (PMC13512694; doi:10.3390/gels12080724)
Supplement: Supplementary file 1 [file gels-12-00724-s001.zip › gels-4378446-supplementary.pdf]

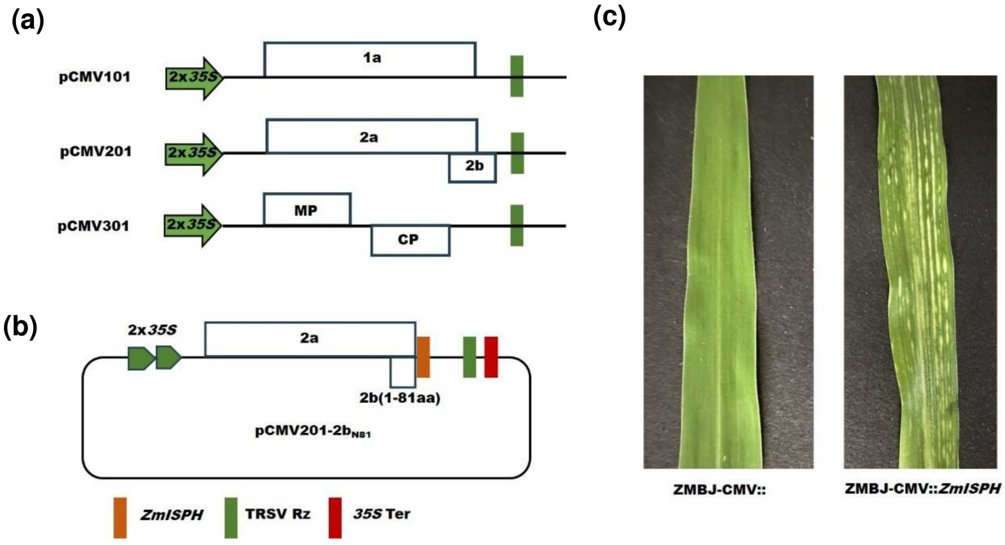

Figure S1. Construction of the ZMBJ-CMV VIGS vector and its functional validation through silencing of *ZmISPH* in maize. (**a**). Schematic diagram of ZMBJ-CMV-based VIGS vectors; (**b**). Construction of the pCMV201-2bN81 vector; (**c**). Photobleaching (chlorosis) phenotypes observed in maize leaves following *ZmISPH* silencing.

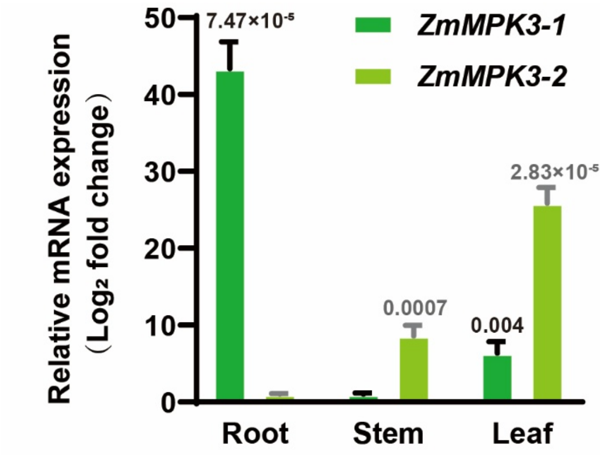

Figure S2. Real-time PCR analysis of *ZmMPK3-1* and *ZmMPK3-2* expression in root, stem and leaf of maize plants. The results were normalized to *ZmUBQ*. Significance of *ZmMPK3-1* and *ZmMPK3-2* expression in different tissues are shown relative to root and stem, respectively. Error bars represent standard errors with three biological replicates and p values are shown on the graph.

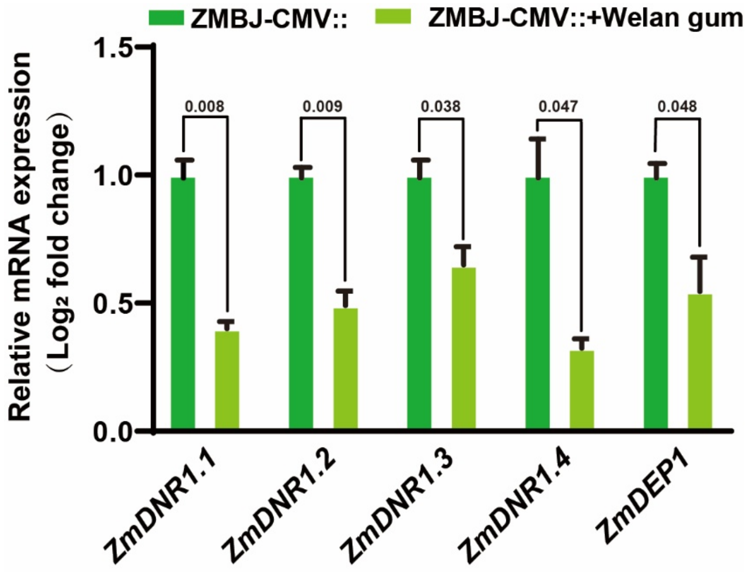

Figure S3. Real-time PCR analysis of *NUE* genes in ZMBJ-CMV-treated maize subjected to welan gum treatment. Real-time PCR results were normalized to *ZmUBQ* and are shown relative to the levels in the empty vector control group. Error bars represent standard errors from three biological replicates and *p* values are shown on the graph.

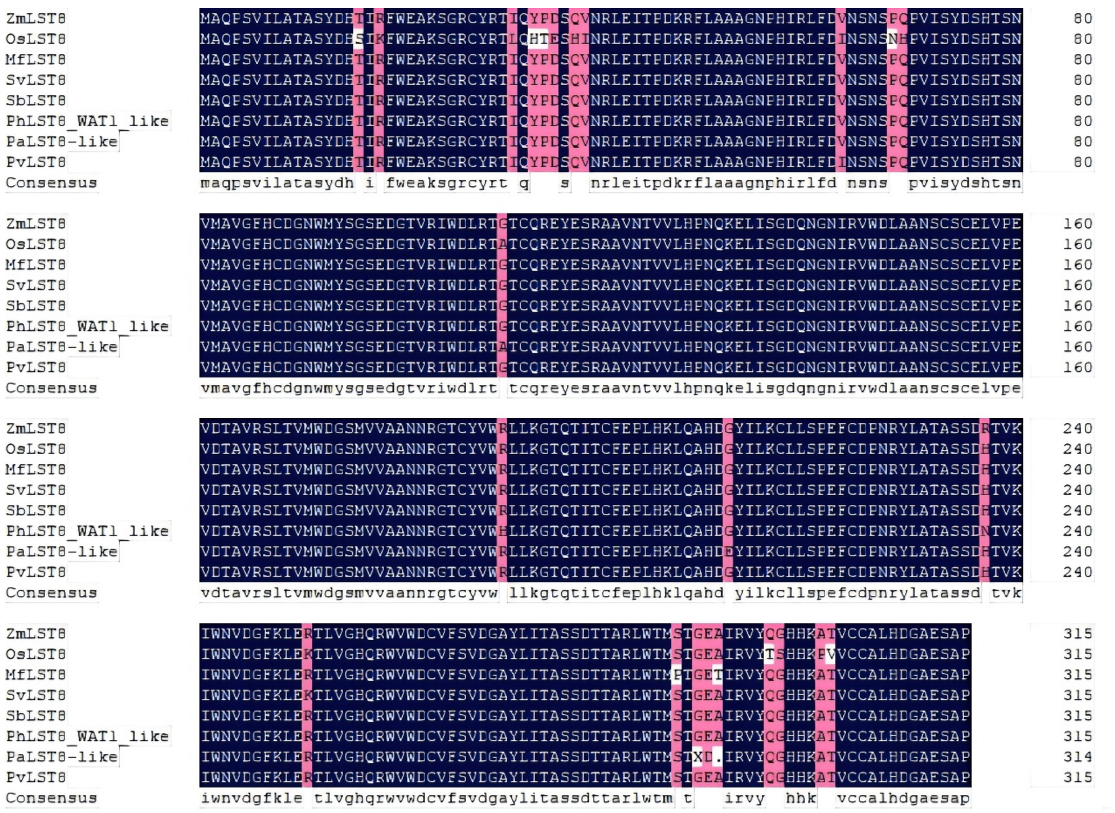

Figure S4. Multiple alignment of LST8 among various cereal plants. ZmLST8, *Zea mays* LST8; OsLST8, *Oryza sativa* LST8; MfLST8, *Miscanthus floridulus* LST8; SvLST8, *Sorghum virgatum* LST8; SbLST8, *Sorghum bicolor* LST8; PhLST8_WAT1_like, *Panicum Hallii* LST8_WAT1_like; PaLST8-like, *Phragmites australis* LST8-like; PvLST8, *Panicum virgatum* LST8.

Table S1 Comparative physicochemical properties of welan gum produced by strain Y503 and a commercial reference

Parameters	Extracted Welan Gum	Commercial reference
**Production & Yield**		
— EPS yield (g/L)	25.42 ± 0.85	—
**Composition (%)**		
— Total sugar content	86.75 ± 1.24	90.52 ± 0.74
— Protein content	2.84 ± 0.31	1.42 ± 0.18
— Nucleic acid residue	0.45 ± 0.08	0.21 ± 0.05
**Elemental Analysis (%)**		
— Carbon (C)	39.25 ± 0.52	39.88 ± 0.42
— Hydrogen (H)	6.41 ± 0.19	6.21 ± 0.19
— Nitrogen (N)	0.58 ± 0.09	0.35 ± 0.08
— Sulfur (S)	ND	ND
**Macromolecular Parameters**		
— $M_w/10^6$Da	1.18	1.15
— Polydispersity index (PDI)	2.30	2.35

The mass balance deficit (approx. 10%) is attributable to tightly bound hygroscopic moisture and trace inorganic salts, as is typical for highly hydrophilic lyophilized polysaccharides. Data are presented as mean ± standard deviation (n = 3). "ND" denotes not detected; "—" indicates not applicable. Commercial reference data are cited from literature.

Table S2 List of primers used for real-time PCR analysis

Primer names	Genes	Primer sequences (5′-3′)
ZmMPK1-F	GRMZM2G048455_T01	TCTCACATGAACTGAGGGGC
ZmMPK1-R		TGGGCTGAATTTTGCCGATTT
ZmMPK2-F	GRMZM2G062914_T03	TGACGGGTCATGGTCACATC
ZmMPK2-R		CCTCCCTTCCTTTCCGTGTC
ZmMPK3-1-F	GRMZM2G053987_T02	TATTACGCGCTGTGCAGGAT
ZmMPK3-1-R		ATGGTTGCCAGGTGTTAGGG
ZmMPK3-2-F	GRMZM2G017792_T01	TCTAGAGCTCGCCAACCACT
ZmMPK3-2-R		TTTTCCTGGACAGCGCGAAT
ZmMPK4-F	GRMZM2G127141_T01	ACCATCCTTTACAGAAGCGCA
ZmMPK4-R		GCAAGCCATTCCCAAGTGAC
ZmMPK6-1-F	GRMZM2G002100_T01	TATCCGCCAACTTCCCTGTC
ZmMPK6-1-R		TGGACAAGCCTTCAAAAGTGT
ZmMPK6-2-F	GRMZM2G020216_T01	TCCCTTGAAGGCTTGTCCATC
ZmMPK6-2-R		AGGCATGCGTTTTCAGACCA
ZmMPK8-F	GRMZM2G375975_T01	CACCTCGGTAACCTCGTACC
ZmMPK8-R		ATCCGGATTCGCATTGTGGA
ZmMPK12-F	GRMZM2G123886_T01	CGTCGATCTGGTTGTCAAACG
ZmMPK12-R		TTCGTTCGGGCTTCACTACT
ZmMPK15-F	GRMZM2G062761_T01	TGTCTGTGCATGACAGTGGG
ZmMPK15-R		CGGTTTTGATGAGAACCTTGGC
ZmMPK16-F	GRMZM2G089484_T01	CTCGTTGGTTGGGTGATCCG
ZmMPK16-R		CGCGAGCTCGTAAGAAAGGT
ZmMPK17-1-F	GRMZM2G374088_T03	CCGTGCTGTCCATTTGCTTG
ZmMPK17-1-R		AGCCGGAACCAATCCAATCC
ZmMPK17-2-F	GRMZM2G306028_T02	CTTTGCATCCGCCAATCTGC
ZmMPK17-2-R		CGCCGTCAGTTACTGTTCCT
ZmMPK17-3-F	GRMZM2G135904_T02	TGCTACTGCTACCGTGCTAC
ZmMPK17-3-R		CGAGTCTGCGACTGAGCAA
ZmMPK18-1-F	GRMZM2G122335_T02	CGCCCATCATTTTCCGTTCT
ZmMPK18-1-R		TTCTGTCCTTACTCGCGGAC
ZmMPK18-2-F	GRMZM2G007848_T01	CCGAGAGGTTATCTGTTGGATTTGC
ZmMPK18-2-R		ACAGTTGGAAAAGTGGCAAAGGATG
ZmMPK19-F	GRMZM2G034052_T01	TTATTGACATCCCCTGTGCCT
ZmMPK19-R		AGTGAAATCAGCGCTAGCAGA
ZmMPK20-1-F	GRMZM2G163861_T01	TCTGCACACGGTTACTGCAT
ZmMPK20-1-R		GTTTGGGAAACCGAAACGGG
ZmMPK20-2-F	GRMZM2G131334_T02	GATCTCTCCCCCGTTTTCCG
ZmMPK20-2-R		GGCACGGAATGCAAAAGAGG
ZmDNR1.1-F	Zm00001eb122460	ATTGTTCGGACGAGAGCTGG

ZmDNR1.1-R		GCTCCGTACGTCAGGACTTT
ZmDNR1.2-F	Zm00001eb336890	CAAAGACGGGAGGTGGACAA
ZmDNR1.2-R		AACCAGCATGGCAGGATGTG
ZmDNR1.3-F	Zm00001eb282730	CGCCGCTGAAAGAAACGATG
ZmDNR1.3-R		ACATTCGAAGCTCCCAGCAT
ZmDNR1.4-F	Zm00001d018715	ACATCGGGTCTGTTCTGTGG
ZmDNR1.4-R		AGTCCCACAGGTGAATGTTTTCT
ZmDEP1-F	Zm00001d005824	CTGTGCCACTGGATGCTTGT
ZmDEP1-R		CGAACGAAACTAACGGTCATCT
ZmTOR-F	Zm00001d037950	AGTGGAGCGGCAGATTAA
ZmTOR-R		ACTGTTGACGCATTTTCTG
ZmLST8-F	Zm00001d007577	GACGCAATTCAGCACAAACC
ZmLST8-R		AGCGCAAGTAAGGGAGGGA
ZmRAPTOR-F	Zm00001d052327	TGATGGCTCCATGGCTTTCG
ZmRAPTOR-R		TCCAAGTTCAACGGCAGGAT
ZmS6K-F	Zm00001d022434	CATCATCAACCGATCCCAT
ZmS6K-R		GACAAGTTCCGATGATCCCT
ZmNRT1.1A-F	GRMZM2G161459	CCGGCGAGAGAAGGAACATT
ZmNRT1.1A-R		AGCTCCACACCTAGGGTCAT
ZmNRT1.1B-F	Zm00001eb023600	CATCCCCAACCCCTCATC
ZmNRT1.1B-R		GCGCACTTGTCTTTCTCAGC
ZmNRT1.6C-F	GRMZM2G112039	AGCGAGCTACCACTACCTA
ZmNRT1.6C-R		CTCATCTCCACATCCATTC
ZmPTR1.2-F	GRMZM2G495015	GGATGGCTTCTTGATAACCT
ZmPTR1.2-R		GCTGGTTTGGTGATGGTT
ZmPTR2.1-F	GRMZM2G085411	GCCGACCCGAATCAATATG
ZmPTR2.1-R		TCTGAGAGCTGCTCCTCCA
ZmPTR3C-F	GRMZM2G375113	CATGTTTGCCAATTTGTGG
ZmPTR3C-R		ACTGATTTAGTTGTGCGTTACC
ZmPTR4.2-F	GRMZM2G018353	GTTGGTACAAGTACAAGCCTCAG
ZmPTR4.2-R		AAGCTTGCACATCTAACAAGGT
ZmPTR6.3-F	GRMZM2G086496	TCCTCCCGAATCCTCACAC
ZmPTR6.3-R		TCCCCTCCCTCTCTCTCTCT
ZmWRKY33-F	LOC100275623	CCCATTTATGCCCCCGAGAG
ZmWRKY33-R		CACGGATCATGTGAACGGGA
ZmERF6-F	LOC100282305	TCAGCTGACGACTTTGCAGT
ZmERF6-R		CAGATCTACTCCGTCTCGGC
ZmPAD3-F	LOC100194166	GAGGACGTGAGCATGGAAGA
ZmPAD3-R		CCACCACAAGATTGACCAGT
ZmIGMT1-F	GRMZM5G814904	TGGTTATTGCGCTGCTACCT
ZmIGMT1-R		TACTCGCACATGGCAGAGAC
ZmUBQ-F	NM_001138130	GCAGTGCTGCAGTTCTACAAGG
ZmUBQ-R		GCAGTGCTGCAGTTCTACAAGG

Table S3 List of primers used for VIGS vector construction

Primer names	Primer sequences（5′-3′）
ZmMPK3-1-VIGS-F-Xba I	GCTCTAGAGCGCACGGTGTTTGTAAATAGGC
ZmMPK3-1-VIGS-R-Kpn I	GGGGTACCCCAGGATGGTTGCCTTGGTTC
ZmLST8-VIGS-F-Xba I	GCTCTAGAGCGACGCAATTCAGCACAAACC
ZmLST8-VIGS-R-Kpn I	GGGGTACCCCAGCGCAAGTAAGGGAGGGA

Table S4 Primer sequences used for vector construction in the Y2H assay

Primer names	Primer sequences（5′-3′）
ZmLST8-F-*Eco*RI	GGAATTCCGACGCAATTCAGCACAAACC
ZmLST8-R-*Bam*H Ⅰ	CGGATCCGAGCGCAAGTAAGGGAGGGA
ZmMPK3-1-R-*Nde*I	GCCATATGATGGCCAACTGCGACCTCAA
ZmMPK3-1-F-*Bam*H I	GCGGATCCGTTTAGGCGGAGTGGATGTACTTGAGC
ZmMPK3-1Δ-F- *Nde*I	GCCATATGATGGCAAATTGTGACCTCAAGA
ZmMPK3-1Δ-R- *Bam*H I	GCGAATTCTTAGTAGGGAGGCTCTGGGTTA
ZmTOR$_{FRB}$-F- *Eco*RI	CCCATATGATGATGACTCTTTGGTTCAACCATGGGG
ZmTOR$_{FRB}$-R-*Xba* Ⅰ	CGGAATTCTCAAATCGTCACAAGTGGTGAATCTGCT

Table S5 Primer sequences for vector construction used in the BiFC assay

Primer names	Primer sequences（5′-3′）
ZmLST8-F-*Bam*H Ⅰ	GCGGATCCATGGCTCAACCTTCAGTCATTCTA
ZmLST8-R-*Xba* Ⅰ	GCTCTAGATTATGAGGGCGCTGATTCTGCT
ZmMPK3-1-F-*Bam*H Ⅰ	CGGGATCCCGGCACGGTGTTTGTAAATAGGC
ZmMPK3-1-R-*Xba* Ⅰ	GCTCTAGAGCAGGATGGTTGCCTTGGTTC
ZmTOR$_{FRB}$-F-*Bam*H Ⅰ	CGGGATCCATGACTCTTTGGTTCAACCATGGGG
ZmTOR$_{FRB}$-R-*Xba* Ⅰ	GCTCTAGATCAAATCGTCACAAGTGGTGAATCTG C

Table S6 Maize growth attribute under rapamycin treatment

Parameters	Control	Welan (100.0 mg/L)	Rapamycin (5.0 μM)	Rapamycin (5.0μM) +Welan (100.0 mg/L)
**Height (cm)**	42.76±4.37b	50.03±3.88c	35.83±4.23a	37.45±1.28a
**Shoot weight (FW/g)**	4.83±0.36b	5.23±0.35c	3.77±0.24a	3.98±0.58a
**Root weight (FW/g))**	2.38±0.31b	2.78±0.43c	1.95±0.35a	2.11±0.31a
**Shoot weight (DW/g)**	0.45±0.05b	0.57±0.09c	0.38±0.01a	0.40±0.01a
**Root weight (DW/g)**	0.24±0.009a	0.34±0.07b	0.20±0.02a	0.23±0.01a

Data are presented as mean ± standard error (n = 10). Different lowercase letters denote statistically significant differences among treatments ($p < 0.05$; one-way ANOVA followed by Duncan's multiple range test; a: $p < 0.05$, b: $p < 0.01$, c: $p < 0.001$). Maize seedlings were exposed to treatments for 10 days under controlled growth conditions.

Table S7 PDBePISA interface results between the selected proteins

**Structure 1**	**Structure 2**	**Interface area (A^2)**	**ΔiG (kcal/mol)**	**ΔiG (*p* value)**
ZmMPK3-1	ZmTOR	3317.1	-21.0	0.181
	ZmLST8	3767.0	-28.8	0.148
	ZmRAPTOR	2312.6	-10.2	0.456

To assess potential interaction interfaces, PDBePISA was used to calculate interface contact areas and the estimated binding free energy (ΔiG) for ZmMPK3-1 paired with ZmTOR, ZmLST8, or ZmRAPTOR.

Table S8 Predicted Subcellular Localization of Selected Proteins

Protein	Subcellular localization
ZmMPK3-1	Nucleus
ZmTOR	Chloroplast; cytoplasm; nucleus
ZmLST8	Nucleus
ZmRAPTOR	Chloroplast; cytoplasm; nucleus

Table S9 Effects of *ZmLST8-1* silencing on growth attribute in maize seedlings

Parameters	ZMBJ-CMV::	ZMBJCMV:: *ZmLST8*
**Height (cm)**	33.8±2.56a	30.25±3.40a
**Shoot weight (FW/g)**	2.15±0.43b	1.72±0.27a
**Root weight (FW/g))**	1.56±0.30b	1.29±0.30a
**Shoot weight(DW/g)**	0.25±0.05b	0.21±0.03a
**Root weight (DW/g)**	0.28±0.02a	0.24±0.08a

Data are presented as mean ± standard error (n = 10). Different lowercase letters denote statistically significant differences among treatments ($p < 0.05$; Student's *t*-test). Maize seedlings were exposed to treatments for 10 days under controlled growth conditions.
